# A Conserved Homeobox Transcription Factor Htf1 Is Required for Phialide Development and Conidiogenesis in *Fusarium* Species

**DOI:** 10.1371/journal.pone.0045432

**Published:** 2012-09-21

**Authors:** Wenhui Zheng, Xu Zhao, Qiurong Xie, Qingping Huang, Chengkang Zhang, Huanchen Zhai, Liping Xu, Guodong Lu, Won-Bo Shim, Zonghua Wang

**Affiliations:** 1 Key Laboratory of Bio-pesticide and Chemistry Biology, Ministry of Education, Fujian Agriculture and Forestry University, Fuzhou, Fujian, China; 2 The Key Laboratory of Sugarcane Biology and Genetic Breeding, Ministry of Agriculture, Fujian Agriculture and Forestry University, Fuzhou, Fujian, China; 3 College of Life Sciences, Henan University of Technology, Zhengzhou, Henan, Fujian, China; 4 Department of Plant Pathology and Microbiology, Texas A&M University, College Station, Texas, United States of America; University of Wisconsin – Madison, United States of America

## Abstract

Conidia are primary means of asexual reproduction and dispersal in a variety of pathogenic fungi, and it is widely recognized that they play a critical role in animal and plant disease epidemics. However, genetic mechanisms associated with conidiogenesis are complex and remain largely undefined in numerous pathogenic fungi. We previously showed that Htf1, a homeobox transcription factor, is required for conidiogenesis in the rice pathogen *Magnaporthe oryzae*. In this study, our aim was to characterize how Htf1 homolog regulates common and also distinctive conidiogenesis in three key *Fusarium* pathogens: *F. graminearm*, *F. verticillioides*, and *F. oxysporum*. When compared to wild-type progenitors, the gene-deletion mutants in *Fusarium* species failed to form conventional phialides. Rather, they formed clusters of aberrant phialides that resembled elongated hyphae segments, and it is conceivable that this led to the obstruction of conidiation in phialides. We also observed that mutants, as well as wild-type Fusaria, can initiate alternative macroconidia production directly from hyphae through budding-like mechanism albeit at low frequencies. Microscopic observations led us to conclude that proper basal cell division and subsequent foot cell development of macroconidia were negatively impacted in the mutants. In *F. verticillioides* and *F. oxysporum*, mutants exhibited a 2- to 5- microconidia complex at the apex of monophialides resulting in a floral petal-like shape. Also, prototypical microconidia chains were absent in *F. verticillioides* mutants. *F. graminearum* and *F. verticillioides* mutants were complemented by introducing its native *HTF1* gene or homologs from other *Fusarium* species. These results suggest that *Fusarium* Htf1 is functionally conserved homeobox transcription factor that regulates phialide development and conidiogenesis via distinct signaling pathways yet to be characterized in fungi.

## Introduction

Asexual sporulation is the preferred mode of reproduction in most pathogenic fungi [1], [2]. More importantly, these asexual spores, commonly known as conidia, are used as a primary dissemination tool as well as for initiating infection [3]–[7]. Under favorable conditions, fungal pathogens can rapidly propagate and spread to cause diseases in economically important crops as well as humans and animals. Significantly, recent studies have shown that fungal pathogens responsible for plant diseases can also cause opportunistic mycosis in humans [8], [9]. However, the mechanisms of asexual sporulation are diverse and complex and remain largely undefined in numerous pathogenic fungi. In model organisms, namely *Neurospora crassa* and *Aspergillus nidulans*, signaling pathways that regulate conidiation have been extensively studied, and excellent reviews are available [2], [10], [11]. Briefly, there are key transcriptional regulators known to be involved in this process. One important gene that plays a critical role in the transition from conidiophore to condia formation is *brlA* in *A. nidulans* [12]. Further genetic and biochemical studies led to the discovery of *abaA* and *wetA* [13], [14]. These three genes (*brlA*-*abaA*-*wetA*) have been proposed to constitute a central regulatory pathway that acts in concert with other genes to control conidiation in *Aspergillus* [15], [16]. However, we also need to recognize that different fungal species may have developed different regulatory mechanisms for producing various types of conidia.

The genus *Fusarium* is considered the most important and diverse genera of plant pathogenic fungi and causes a wide range of diseases in every economically important crop species [17], [18]. Several species within the genus are also associated with the production of mycotoxins which poses a significant threat to food safety and human health [19]–[22] Moreover, once considered a relatively uncommon cause of ocular disease, *Fusarium* species have emerged as one of the leading causes of human keratomycosis outbreaks, along with *Aspergillus* and *Candida* species [23], [24] The genomes of closely related *Fusarium* species, *F. graminearum*, *F. verticillioides*, and *F. oxysporum*, have been sequenced mainly due to their economic and scientific importance [18], [25], [26]. In addition, these *Fusarium* species offer a unique opportunity to investigate numerous biological features, including distinct asexual sporulation modes. In recent years, a number of conidia-related genes in *Fusarium* species have been identified by insertional mutagenesis or targeted gene deletion approaches. Several genes are important transcriptional regulators, such as *FgSTUA*, *FoSTUA* and *REN1*, which are conserved in filamentous fungi and essential for conidiogenesis [27]–[29]. Genes such as *FgVEA*, *FvVE1* and *FgTEP1* are involved in multiple signaling pathways, regulating virulence, secondary metabolism and conidiation [30]–[32]. Some important signal transduction related genes, e.g., *GPMK1*, *GzSNF1* and *FAC1*, which encode various protein kinases are also required for conidiation in *Fusarium* species [33]–[37]. However, molecular mechanisms underlying conidiogenesis in *Fusarium* species is complex and does not seem to adhere to the regulatory pathway established in *A. nidulans* and *N. crassa* [2], [10], [11], [38]. Therefore, it is currently difficult to unambiguously define genetic mechanisms or signaling pathways required for this important biological process in Fusaria.

Although *F. graminearum*, *F. verticillioides* and *F. oxysporum* are defined into the same genus, they exhibit distinct features in asexual sporulation. For instance, *F. graminearum* only produces macroconidia on solitary phialides or on multiple phialides borne on conidiophores [27]. In *F. verticillioides*, the fungus grows as haploid mycelia and propagates vegetatively via hyphal elongation and produces two types of asexual spores, macroconidia and microconidia [39]. Macroconidia emerge from macroconidiophores, which are branched and unbranched monophialides [40], [41]. Similarly, unicellular and uninucleate microconidia also arise from branched and unbranched monophialides, frequently forming long conidial chains and false heads. When compared to the other two, *F. oxysporum* is unique in the fact that it can only reproduce asexually, but through three different types of conidia: microconidia, macroconidia, and chlamydospores [40]–[42]. Microconidia are ellipsoidal and have no or one septum, macroconidia are falcate and have three or four septa, and chlamydospores are globose-like with thick walls [29], [40].

Despite the structural difference between macroconidia and microconidia, conidiation pattern is very similar, and the use of enteroblastic mechanisms from the phialide is common in all three species [43]. In *Fusarium* species, phialides are cylindrical, solitary or produced as a component of a complex branching system. Microconidia are formed from phialides with false heads or from long chains by basipetal division, from the apex toward the base [44], [45]. Macroconidia with pronounced foot cells are generally produced from phialides on conidiophores also by basipetal division [46]. However, in *F. oxysporum* the chlamydospores are produced acrogenously from hyphae or by the modification of hyphal cells and conidial cells through the condensation of their contents [47].

Transcriptional regulation plays a critical role in altering the expression of specific subsets of genes associated with development and differentiation in cells [48], [49]. In our previous study, we identified and characterized Htf1, an important homeobox transcription factor (TF) required for conidiogenesis, in *Magnaporthe oryzae* [7], [50]. We reasoned that Htf1 may also play a critical role in *Fusarium* conidiation. However, considering that different *Fusarium* species have different mode of conidiogenesis, we also hypothesized that Htf1 can perform unique function in different species. In this study, our aim was to functionally characterize Htf1 orthologs in *F. graminearum*, *F. verticillioides* and *F. oxysporum*, and determine its role in conidiogenesis. Results showed that Htf1 ortholologs in three *Fusarium* species have significant similarity in specifically regulating phialidegenesis and subsequent macroconidiation. Moreover, Htf1 ortholologs in *F. verticillioides* and *F. oxysporum* are required for morphogenesis of microconidial chains and false heads.

## Results

### Comparative analysis of Htf1 orthologs in *Fusarium* species

**Figure 1:**
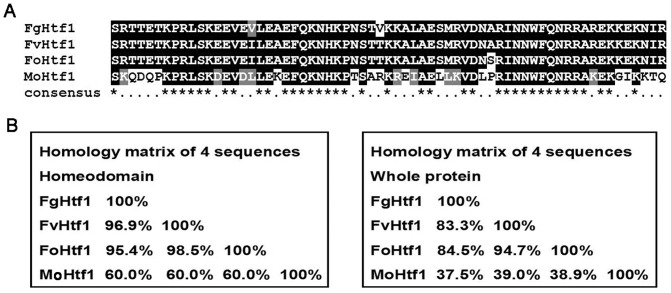
Comparative analysis of Htf1 protein in three *Fusarium* species. (A) Sequence alignment of the homeodomain in Htf1 homolog of *F. graminearum* (FgHtf1), *F. verticillioides* (FvHtf1), *F. oxysporum* (FoHft1) and *M. oryzae* (MoHtf1) was performed using Clustal W and Boxshade (http://bioweb.pasteur.fr/seqanal/interfaces/boxshade.html). The conserved amino acid residues are shaded black, whereas similar residues are shown in gray. Consensus amino acids are marked with asterisk (*). (B) Homology matrix analysis of Htf1 homeodomain (left box) and whole protein (right box) in three *Fusarium* species and *M. oryzae* by DNAMAN software. Numbers (%) indicate protein identity.

Htf1 was reported previously as a key regulator of conidiogenesis in *M. oryzae* [7], [50]. To investigate functional conservation, we first isolated genes encoding Htf1 homolog in three *Fusarium* species as well as other filamentous fungi via BLAST analysis. Study of databases (Broad Institute Fungal Genome Initiative [www.broadinstitute.org] and Fungal Transcription Factor Database [ftfd.snu.ac.kr]) revealed that *M. oryzae* Htf1 homolog is present in these fungal species and that these homologs contain a conserved homeodomain motif, predominantly located in the N-terminus region (data not shown). As anticipated, the homeodomain motif was highly conserved in *Fusarium* species and *M. oryzae* (Figure 1A), but a high level of variability was observed in the C-terminus region between *M. oryzae* and *Fusarium* species. When we compared the predicted protein sequence of *F. graminearum* FGSG_07097 gene (designated *FgHTF1*), *F. verticillioides* FVEG_08072 gene (designated *FvHTF1*), *F. oxysporum* FOXG_01706 gene (designated *FoHTF1*), and *M. oryzae* MGG_00184 gene (previously *HTF1* [50], designated *MoHTF1* in this manuscript), identity was greater than 95% at the protein level within the homeodomain amongst *Fusarium* species, and MoHtf1 homeodomain shared 60% identity with *Fusarium* counterparts (Figure 1B). These genes also share a highly conserved exon/intron structure (Figure S1A). Outside the homeobox domain region, protein similarity drops significantly when compared across fungal species (data not shown), and no known functional motifs or biologically significant domains exist (Figure S1B). In *Arabidopsis thaliana* Athb-12, the C-terminus region has been shown to serve as the activation domain [51], but functional role of Htf1 C-terminus region in fungi have not been characterized to date.

### *FgHTF1* is dispensable for vegetative growth and fertility but essential for conidiation

In order to study the function of Htf1 ortholog in *Fusarium* conidiogenesis, we first deleted *FgHTF1* in *F. graminearum* using gene replacement approach (Table S1, Figure S2A). Transformants were selected on hygromycin-amended medium, and gene deletion was confirmed by polymerase chain reaction (PCR) and Southern blot analyses (Figure S2B and S2C). *FgHTF1* deletion mutant (*ΔFghtf1*) showed no discernible difference in vegetative growth and sexual reproduction when compared to the wild-type strain PH-1 on complete medium (CM) and wheat kernels medium, respectively (Table 1, Figure S3A and S3B). However, *ΔFghtf1* mutant showed significantly reduced macroconidia production in liquid carboxymethylcellulose (CMC) medium [52]. After three days, only 1.00±0.87×10^4^ macroconidia were observed in *ΔFghtf1*, whereas 51.44±6.64×10^4^ macroconidia were in PH-1 (Table 1). Even after fifteen days of incubation, *ΔFghtf1* mutant did not recover the production of macroconidia compared to PH-1, indicating that this reduction in conidiation was not related to the duration of incubation (Table 1). The defect in conidia production was fully recovered to the wild-type level in the complemented strain *ΔFghtf1-Com*, where the native promoter-driven *FgHTF1* gene cassette was re-introduced into *ΔFghtf1* (Figure S2B and S2C, Table 1). These results indicate that *FgHTF1* is critical for macroconidia production in *F. graminearum*.

**Table 1:** Characterization of *ΔFghtf1* and complementation transformants.

Strain	Growth (cm)^a^	Conidiation 3 d (10^4^/ml)^b^	Conidiation 6 d (10^4^/ml)^b^	Conidiation 15 d (10^4^/ml)^b^	Phialide cell	Germination (%)^c^	Foot cell of conidium	Wheat disease level^d^
PH-1	6.10±0.04	51.44±6.64	151.00±6.36	184.56±6.82	normal	98.89±1.39	normal	8.11±1.90
*ΔFghtf1*	6.12±0.07	1.00±0.87	4.11±1.45	6.50±1.41	abnormal	97.91±2.52	lost	7.77±2.28
*ΔFghtf1-Com*	6.10±0.04	52.89±5.60	148.22±6.70	187.11±10.19	normal	98.96±1.67	normal	7.67±1.73
*ΔFghtf1-Fv*	6.14±0.04	55.56±4.61	156.89±8.05	185.22±10.33	normal	98.64±1.87	normal	nd^e^
*ΔFghtf1-Fo*	6.16±0.05	58.67±5.98	154.89±6.94	185.33±12.89	normal	98.41±1.64	normal	nd

a Radial growth was measured as the diameter of colonies after 3 days incubation on complete agar medium. Means and standard errors were calculated from three independent experiments.

b Number of spores/ml after given days of growth on carboxymethylcellulose media.

c Percentage of conidial germination was measured under a light microscope after incubation in liquid CM for 2 h. The experiments were replicated three times, and more than 100 conidia were observed each time.

d Disease was rated by the number of symptomatic spikelets 14 days after inoculation of a basal spikelet.

e nd = Not detected.

### *FgHTF1* specifically regulates phialidegenesis and subsequent conidiation

**Figure 2:**
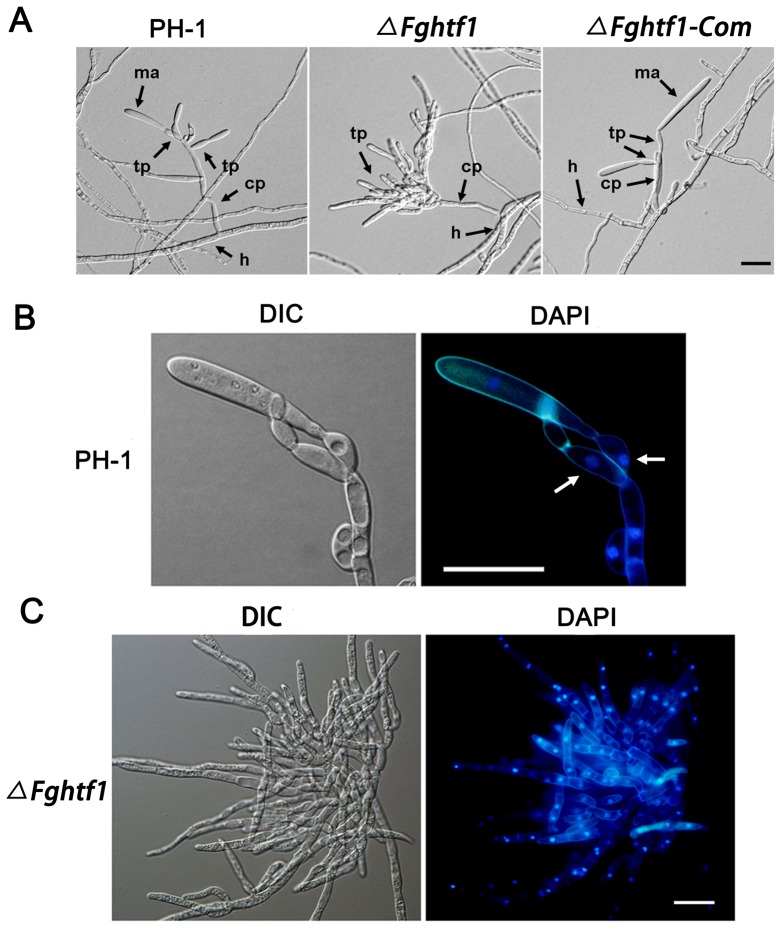
FgHtf1 regulates the differentiation of phialides and subsequent macroconidiation. (A) Wild-type strain PH-1 and complementation strain *ΔFghtf1-Com* produced abundant macroconidia borne on terminal phialides while the *ΔFghtf1* mutant produced aberrant terminal phialides but failed to form macroconidia. tp, terminal phialides; ma, macroconidia; cp, conidiophore; h, hyphae. Bar = 20 µm. (B) Fluorescence staining of nuclei with DAPI demonstrated that phialide-like structures in wild type were uninucleate (white arrow). Bar = 20 µm. (C) Fluorescence staining of nuclei with 4′6-diamidino-2-phenylindole (DAPI) observes clumps consisting of hyphal segments in *ΔFghtf1* mutant. Bar = 20 µm.

In order to investigate the reason for significantly reduced conidiation in *ΔFghtf1*, we microscopically observed fungal tissues grown in CMC, a medium that promotes fungal conidiation. Under the same culture condition, PH-1 and *ΔFghtf1-Com* produced typical conidiogenous cells, *i.e*., phialides, which divide to produce incipient macroconidia. The morphology of phialides in PH-1 and *ΔFghtf1-Com* assumes a bottle-like shape (Figure 2A). The mutants, however, did not produce these structures on its conidiophores, but rather formed clusters consisting of hyphal segments (Figure 2A). Fluorescence staining of nuclei with 4′6-diamidino-2-phenylindole (DAPI) showed that a phialide in the wild type was uninucleate and harbors a macroconidium (Figure 2B). However, in *ΔFghtf1* it appeared that multiple phialide-like structures were disorderly formed on a conidiophore with no macrocondia development (Figure 2C). Presumably, the mutation in *FgHTF1* led to abnormal conidiogenous cells with uncontrolled proliferation and the loss of conidiation capacity. These results suggest that FgHtf1 governs proper differentiation of phialides and is continuously required for maintenance of conidiogenesis in *F. graminearum*.

### *FgHTF1* regulates macroconidia basal cell division and foot cell development

**Figure 3:**
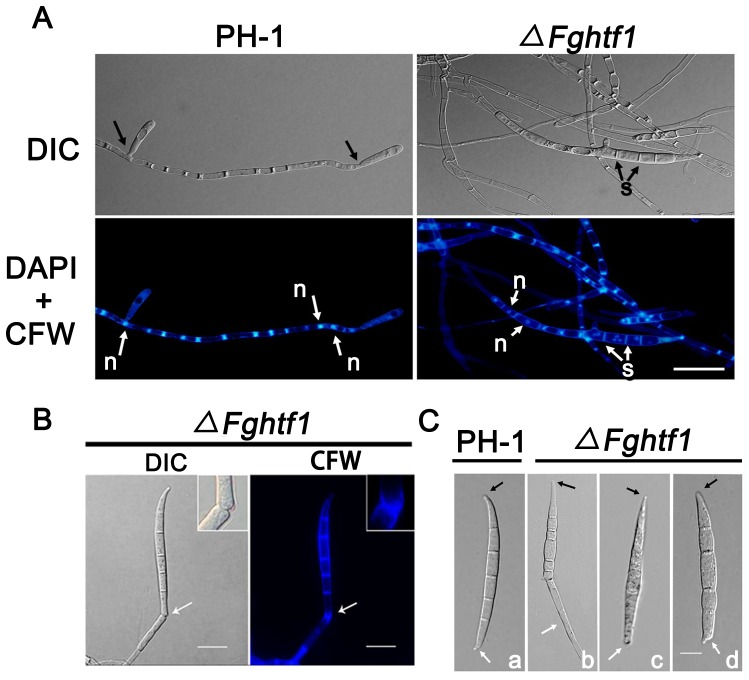
Macroconidial defect in Δ*Fghtf1* mutant. (A) Budding pattern of macroconidial development in *F. graminearum.* Wild-type PH-1 strain produced incipient macroconidium without distinct septation directly from hyphae. *ΔFghtf1* deletion mutant produced matured conidium with clear septation from the tip of hyphae. The resulting cells were observed with a DIC microscope and also stained with DAPI and calcofluor white (CFW) to visualize nuclei and septa, respectively, under a fluorescence microscope. Black arrows in PH-1 strain indicated the position of cell division. s, septa, n, nucleus. Bar = 20 µm. (B) Macroconidia matured and then released from the hyphae by mechanical force during incubation with agitation. *ΔFghtf1* mutant was stained with calcofluor white (CFW) to visualize cell wall and septa. White arrows indicate the point of breakage. Bar = 20 µm. (C) Morphological phenotypes of macroconidia produced by PH-1 and *ΔFghtf1* mutant. a, PH-1, typical *Fusarium* macroconidium, the apical cell is in the below (black arrow), and the foot cell is on the top (white arrows). b–d, *ΔFghtf1* mutant, an obvious defect in foot cell. Bar = 20 µm.

While *ΔFghtf1* deletion mutant lost its ability to produce macroconidia from phialides, which is the main conidiogenesis structure in *F. graminearum*, we still observed some conidia produced in CMC medium, suggesting that alternative conidiation mechanisms exist. After further microscopic observation, we detected *ΔFghtf1* and PH-1 producing spores directly from hyphae, similar to budding observed in *Saccharomyces cerevisiae*, albeit at low frequencies (Figure 3A). However there was also a significant difference in conidiogenesis between *ΔFghtf1* and PH-1. In the early stages of culturing PH-1 (within 48 h), incipient conidium broke off from intercalary or terminal hyphae without distinct septation, whereas in *ΔFghtf1* no conidium was observed at the early stages of culturing in CMC medium. Only after five days of incubation, *ΔFghtf1* produced some matured conidia with evident septation at the tip of hyphae (Figure 3A). In addition, we noticed that there was no recognizable narrow region that allows conidium to detach easily from hyphae, and hence, these conidia lack the typical enteroblastic phenomenon associated with PH-1 (Figure 3A). In PH-1, the narrow region serves as the site for the production of macroconidium by cell division. Therefore, we inferred that the dissociative spores may be ruptured away from *ΔFghtf1* hyphae by mechanical force during shaking incubation (Figure 3B), and this may explain why macroconidia produced by *ΔFghtf1* were morphologically aberrant (Figure 3C). The wild-type macroconidia were moderately curved on the dorsal side and straight on the ventral surface with papillate apical cells and distinct foot-shaped basal cells (Figure 3C). However, *ΔFghtf1* conidia were grotesque without proper foot-shaped basal cells (Figure 3C). These observations indicate that *FgHTF1* is important for proper basal cell division and subsequent foot cell development in macroconidia produced directly from hyphae.

### Aberrant macroconidia of *ΔFghtf1* mutant still can germinate properly and be pathogenic on hosts

**Figure 4:**
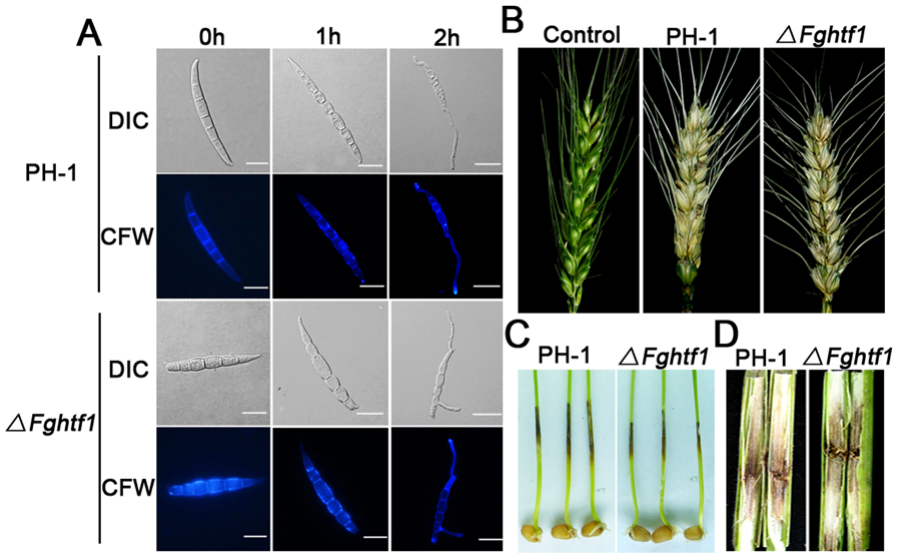
Aberrant Δ*Fghtf1* macroconidia can germinate and are pathogenic to host. (A) Germination of wild-type PH-1 and mutant *ΔFghtf1* macroconidium in CM. The cells were first observed with a DIC microscope, and subsequently stained with calcofluor white (CFW) to visualize cell walls and septas, respectively, under a fluorescence microscope. Bar = 20 µm. (B) Wheat heads were point-inoculated in the two central spikelets each with 200 conidia of PH-1 and *ΔFghtf1*. Control wheat heads were point-inoculated with distilled water. Infection assay was terminated after 21 days. (C) Infected wheat coleoptiles inoculated with conidia of the PH-1 and *ΔFghtf1*. The brown lesions observed on coleoptiles (in length and discoloration intensity) were not distinguishable in PH-1 and *ΔFghtf1* samples. (D) Corn stalks were inoculated with toothpicks carrying a mycelium block of PH-1 and *ΔFghtf1.* Infected corn stalks were split longitudinally at the inoculation sites and examined 14 days post inoculation.

While *ΔFghtf1* produced a limited number of macroconidia by budding and have a defect in the foot cell, these spores were still able to germinate like wild type at 25°C in liquid CM with gentle agitation. After 1 h incubation, approximately 70% of macroconidia in *ΔFghtf1* and PH-1 looked swollen (Figure 4A). After 2 h, over 95% of macroconidia had at least one germ tube from terminal cells, intercalary cells, or both in the mutant (Figure 4A, Table 1). To determine whether *FgHTF1* has a role in pathogenicity, we inoculated wheat heads and wheat coleoptiles with conidia from PH-1 and *ΔFghtf1*. At 14 days post inoculation (dpi), typical ear rot symptoms were observed on wheat head inoculated with PH-1 and the *ΔFghtf1* (Figure 4B, Table 1). Similar brown lesions on coleoptiles and corn stalks infected by PH-1 and *ΔFghtf1* were observed (Figure 4C and 4D). These results showed that the aberrant *ΔFghtf1* macroconidia can germinate properly and be pathogenic on hosts.

### Expression of *FgHTF1* correlates with conidiophore development in *F. graminearum*

**Figure 5:**
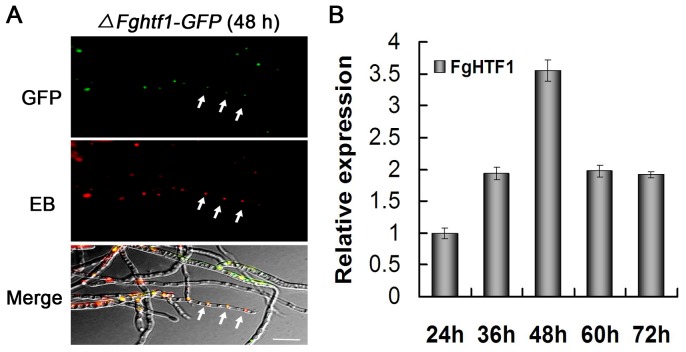
Expression and localization of the GFP-fusion protein. (A) Expression of *FgHTF1* in mycelia of *F graminearum* transformants *ΔFghtf1-GFP* grown in CMC medium, which is conducive to spore production. The strain *ΔFghtf1-GFP* carries a single GFP-carboxy translational fusion of *FgHTF1*. GFP fluorescence was observed in mycelia at 48 h after inoculation, and each cell contained one fluorescence punctum. Mycelia of *ΔFghtf1-GFP* were also stained by ethidium bromide (EB), which is a nuclear counterstain for use in multicolor fluorescent techniques and stains nuclei specifically. The merged image of GFP and EB staining showed that *ΔFghtf1-GFP* localizes to the nucleus (white arrow), Bar = 20 µm. (B) Expression levels of *ΔFghtf1-GFP* at different time course (24 h, 36 h, 48 h, 60 h and 72 h) after inoculation in CMC medium. qRT-PCR was used to quantify transcript level of *FgHTF1* relative to that of the constitutive reference gene β-tubulin using the 2^−*ΔΔ*C^_T_ method.

In order to investigate the temporal and spatial pattern of *FgHTF1* expression during conidiogenesis, *FgHTF1* gene with its native promoter was fused in-frame to the green fluorescent protein (GFP)-encoding gene. The construct was then transformed into *ΔFghtf1* protoplasts. Subsequently, we isolated three transformants expressing GFP in hyphae, and the presence of the FgHtf1-GFP construct was confirmed by PCR (data not shown). All positive transformants (*ΔFghtf1-GFP*) produced a similar number of conidia when compared to the wild-type progenitor. To investigate the expression patterns of FgHtf1 during conidia germination in *F. graminearum*, we followed GFP expression by fluorescence microscopy at different time points (24 h, 36 h, 48 h, 60 h and 72 h) after inoculating *ΔFghtf1-GFP* mycelia into CMC medium, which is conducive to spore production. GFP signals were not detectable or extremely weak from 24 h to 36 h when *ΔFghtf1-GFP* strain was incubated in CMC (data not shown). However, at 48 h GFP signal spiked, and the localization of FgHtf1 to nucleus was verified by GFP and ethidium bromide (EB) stain (Figure 5A). To study expression patterns of *FgHTF1* in PH-1, we extracted total RNA from PH-1 cultured in CMC medium at 24 h, 36 h, 48 h, 60 h and 72 h. Real-time PCR detected a high-level expression of *FgHTF1* at 48 h during the sporulation-induced stage (Figure 5B), but leveled off at 60 h and 72 h. We have observed previously that conidiophores typically proliferate after 48 h time point in CMC culture (data not shown). Thus, we can conclude that FgHtf1 transcription factor is activated and localized to the nucleus prior to conidiogenesis and perhaps regulates expression of genes associated with conidiophore and phialide development in a temporal and spatial specific manner.

### *FvHTF1* regulates microconidia chain and false head formation in *F. verticillioides*

*F. graminearum* only produces macroconidia as asexual reproduction, and to further investigate whether Htf1 plays a role in microconidiation in *Fusarium* species, we generated *FvHTF1* null mutation (*ΔFvhtf1*) in *F. verticillioides*, which produces both macroconidia and microconidia. Homologous recombination that resulted in *FvHTF1* deletion was identified by PCR and was further confirmed by Southern blot (Figure S4A and S4B). *ΔFvhtf1* mutant did not show a significant difference in vegetative growth and microconidia morphology when compared to the wild-type strain A149 grown on various media, including complete medium (CM) and mung bean agar medium (Figure S5A and S5B).

**Figure 6:**
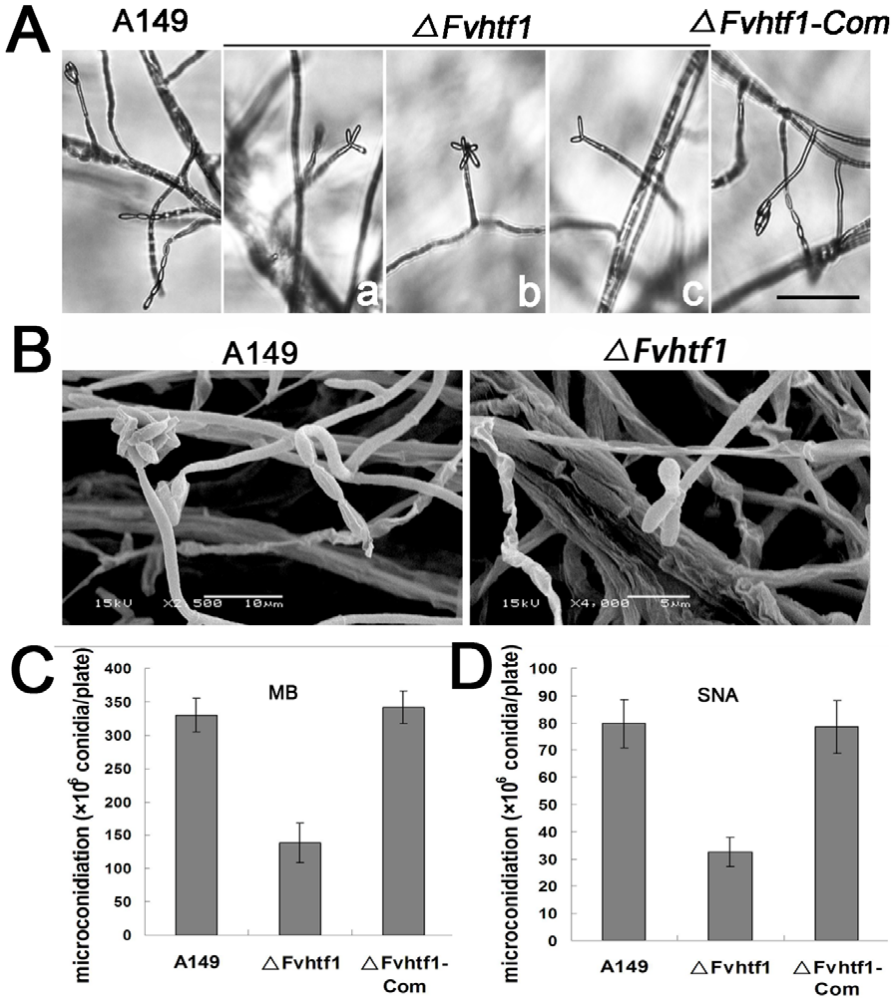
FvHtf1 regulates microconidiation in chain and false head. (A) Light microscope images of microconidiogenesis in wild-type *F. verticillioides* (A149), *FvHTF1* gene-deletion mutant (*ΔFvhtf1*) and complementated strain (*ΔFvhtf1-Com*). Chains and false heads of microconidia were evidently observed in *F. verticillioides* A149 and *ΔFvhtf1-Com*. In *ΔFvhtf1* mutant, microconidial chains were absent and instead microconidia were assembled in a aberrant petal-shaped false heads (a∼c). Bar = 50 µm. (B) Scanning electron microscopy of A149 and *ΔFvhtf1* microconidiation. Wild-type strain A149 showed the typical chains and false heads of microconidia (bar = 10 µm), but *ΔFvhtf1* failed to produce microconidial chains and instead produced false heads with 2 to 5 microconidia at the apex of monophialides resulting in a floral petal shape (bar = 5 µm). (C) Microconidia produced by A149, *ΔFvhtf1*, and *ΔFvhtf1-Com* in a 7-day-old mung bean agar culture. (D) Microconidia produced by A149, *ΔFvhtf1*, and *ΔFvhtf1-Com* in a 7-day-old SNA culture.

When examined under optical and scanning electron microscopes, A149 produced long chains of microconidia and false heads of microconidia aggregates (Figure 6A and 6B), which are customary taxonomic features of *F. verticillioides*. In contrast, we did not find microconidia chains in *ΔFvhtf1* even with repeated efforts on various cultures conditions, even though there was no distinct difference in conidiophore morphology. In addition, microconidia of *ΔFvhtf1* did not stay attached to each other to form false heads as typically observed in the wild-type progenitor (Figure 6A and 6B). In *ΔFvhtf1*, the shape of conidia false head produced from the conidiophore varied, some assumed a *trifolium pretense-*like shape, others looked like petal, and some also possess dichotomization shape (Figure 6A and 6B). Although the shape of microconidia cluster produced by *ΔFvhtf1* varied in their appearances, they displayed a common conidiogenesis pattern, which suggests that all microconidia were verticillate branches at the apex of monophialides (Figure 6A and 6B). To further confirm the microconidial chain and false head shape defect in *ΔFvhtf1*, the mutant strain was transformed with the corresponding wild-type gene. Complementation of *FvHTF1*, where the native promoter-driven *FvHTF1* gene cassette was re-introduced into *ΔFvhtf1*, restored microconidia chains and false head pattern (Figures S4B and 6A), demonstrating that *FvHTF1* is needed for the formation of microconidial chains and false head pattern in *F. verticillioides*.

When we assayed the production of microconidia on 7-day mung bean agar and synthetic low-nutrient agar (SNA) cultures, the wild-type strain produced about twice as much microconidia than *ΔFvhtf1* (Figure 6C and 6D), suggesting that a significant reduction in conidiation could be due to a defect in formation of microconidia chains. It is conceivable that altered microconidiation pattern observed in *ΔFvhtf1* prohibits the fungus from developing long chains of microconidia through basipetal division typically observed in the wild-type *F. verticillioides* [40], [41], [44]. Microconidia production was fully restored to the wild-type level in the complemented strain *ΔFvhtf1-Com* (Figure 6C and 6D).

### The Htf1-regulated macroconidiation is conserved in *F. verticillioides* and *F. graminearum*

**Figure 7:**
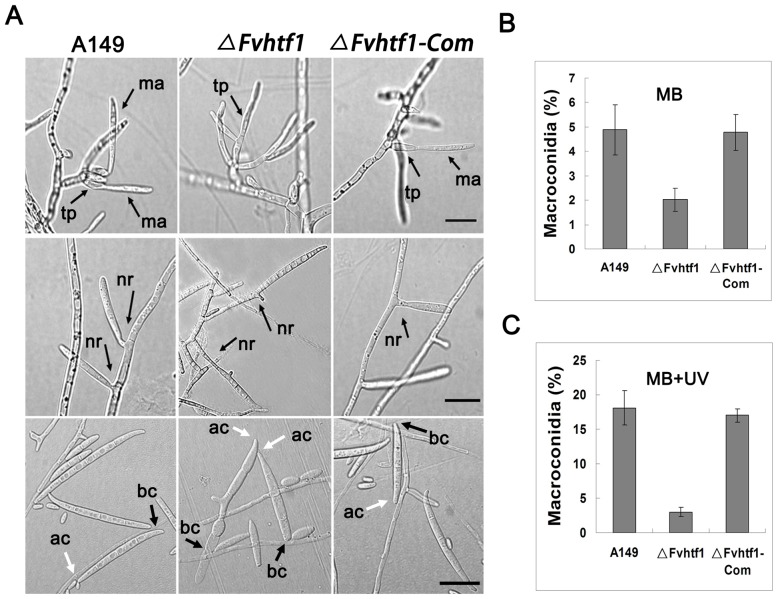
Macroconidiation in *ΔFvhtf1* mutant. (A) In wild-type strain (A149) and the complementation strain (*ΔFvht1-Com*), incipient macroconidia without septation are produced on terminal phialides or hyphae. The gene-deletion mutant (*ΔFvhtf1*) failed to form macroconidia from aberrant terminal phialides. *ΔFvhtf1* produced mature conidium with clear septation from the tip of the hyphae. The macroconidia of *ΔFvhtf1* deletion mutant showed a morphological defect. These observed phenotypes in *ΔFvhtf1* are consistent with what we witnessed in *F. graminearum* mutant (*ΔFghtf1*). tp, terminal phialides; ma, macroconidia; nr, narrow region, ac, apical cell; bc, basal cell (foot cell). Bar = 20 µm. (B) Macroconidia production by A149, *ΔFvhtf1*, and *ΔFvht1-Com* in mung bean liquid medium under continuous dark conditions. (C) Macroconidia production by A149, *ΔFvhtf1*, and *ΔFvht1-Com* in mung bean liquid medium under UV light conditions.

While microconidia are the predominant form of asexual spores in *F. verticillioides*, it also produces macroconidia in nature and in certain laboratory conditions [36], [53]. When we monitored the development process of macroconidiation in *F. verticillioides*, we observed the mechanism similar to *F. graminearum*, in which macroconidia are produced on solitary phialides or on multiple phialides borne on conidiophores (Figure 7A). In *ΔFvhtf1*, we found that the macroconidiogenesis from phialide was impaired (Figure 7A), however, the mutant used budding pattern to produce foot cell-defective macroconidia, which is identical to what we observed in *F. graminearum ΔFghtf1* mutant (Figure 7A).

In addition, we assayed for the amount of macroconidia in mung bean liquid medium under continuous dark and UV light conditions. In continuous dark condition, the wild-type and complemented strains produced a similar level of macroconidia, which typically accounts for approximately 5% of the total conidia harvested from *F. verticillioides* cultures after 7 days. Under the same conditions, however, only 2% of the conidia were macroconidia in the *ΔFvhtf1* mutant (Figure 7B). These data suggested that *FvHTF1* plays an important role in macroconidia development. UV light is known to stimulate macroconidia production in *Fusarium* species [36], [53], and under UV light approximately 17% of the total conidia harvested after 7 days of incubation were macroconidia in wild-type and complemented strains. This is a significant increase when compared to the continuous dark condition. However, in *ΔFvhtf1* mutant the percentage of macroconidia (2%) was consistent with that produced under continuous dark condition (Figure 7C). These results suggested that *FvHTF1* is important for macroconidia production and that it may plays a role in cellular responses to UV light stimulus.

### *FoHTF1* is also required for the development of microconidia and macroconidia, but not for chlamydospores

**Figure 8:**
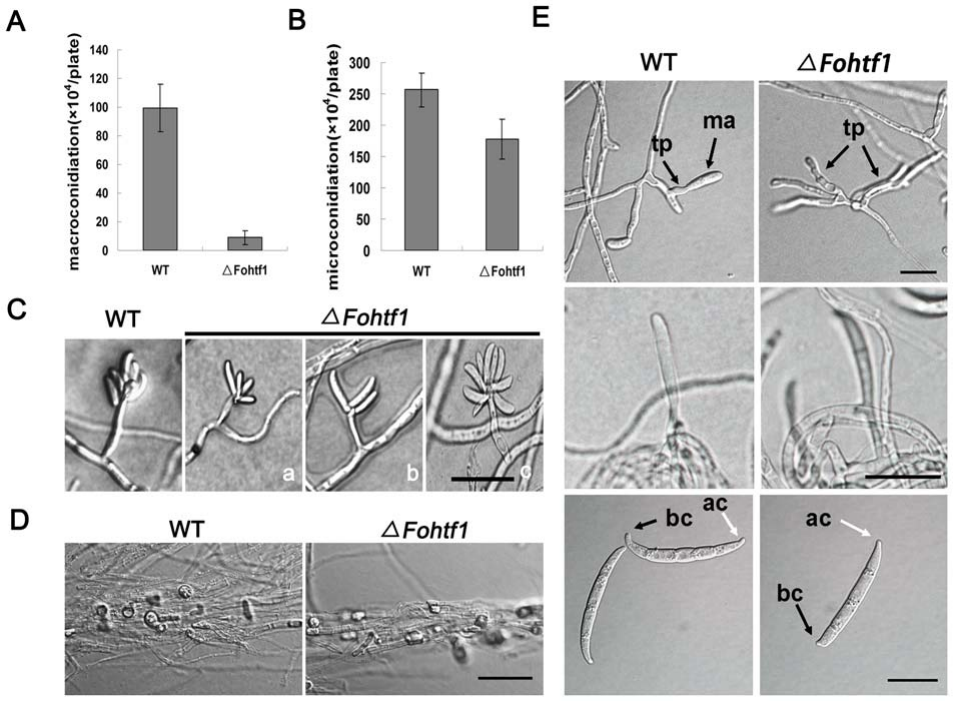
Conidiation in *ΔFohtf1* mutant. (A) The wild-type *F. oxysporum* (WT) and the gene-deletion mutant (*ΔFohtf1*) strains were assayed for macroconidia production in SNA medium under continuous UV light. (B) WT and *ΔFohtf1* strains were assayed for macroconidia production in SNA medium under continuous UV light. (C) WT and *ΔFohtf1* strains were grown on SNA medium for 5 days. In the wild-type strain, microconidia were produced from phialides generally in false heads. *ΔFohtf1* lacked prototypical false head microconidia but rather formed a windmill-shaped microconidia head (a∼c). Bar = 20 µm. (D) Chlamydospores are formed from hyphae of WT and *ΔFohtf1* strains. Bar = 20 µm. (E) Aberrant terminal phialides and macroconidia produce by *ΔFohtf1* mutant. tp, terminal phialides; ma, macroconidia; ac, apical cell; bc, basal cell (foot cell). Bar = 20 µm.

The results we obtained from *F. graminearum* and *F. verticillioides* studies led us to further explored *F. oxysporum* conidiogenesis. This fungus produces three types of asexual spores: microconidia, macroconidia, and chlamydospores. To determine whether the function of Htf1 is conserved in *F. oxysporum* conidiogenesis, we generated a gene-replacement mutant of *FoHTF1* (Figure S6). The mutant (*ΔFohtf1*) was normal in vegetative growth and microconidia morphology (Figure S7), but when assayed for conidiation on SNA medium we found that *ΔFohtf1* had a significant reduction in macroconidia and a slight reduction in microconidia when compared to the wild type (Figure 8A and 8B). The *ΔFohtf1* and the wild-type strain were examined under an optical microscope, and we noticed that the mutants lacked normal false head microconidia and formed windmill-shaped structure, which was congruent with microconidiogensis in *ΔFvhtf1* mutant (Figure 8C). However, *ΔFohtf1* produced normal chlamydospores acrogenously from hyphae or by the modification of hyphal cells, as the wild type (Figure 8D). Phialides of macroconidia also redundantly proliferated and developed constant extension very similar to *ΔFghtf1* and *ΔFvhtf1* mutants (Figure 8E). These results indicate that *FoHTF1* is important for conidiophore and phialide development, and ultimately microconidia and macroconidia production. However we concluded that *FoHTF1* is not involved in hyphal differentiation that leads to chlamydospores in *F. oxysporum.*

### The function of Htf1 is conserved in three *Fusarium* species

**Figure 9:**
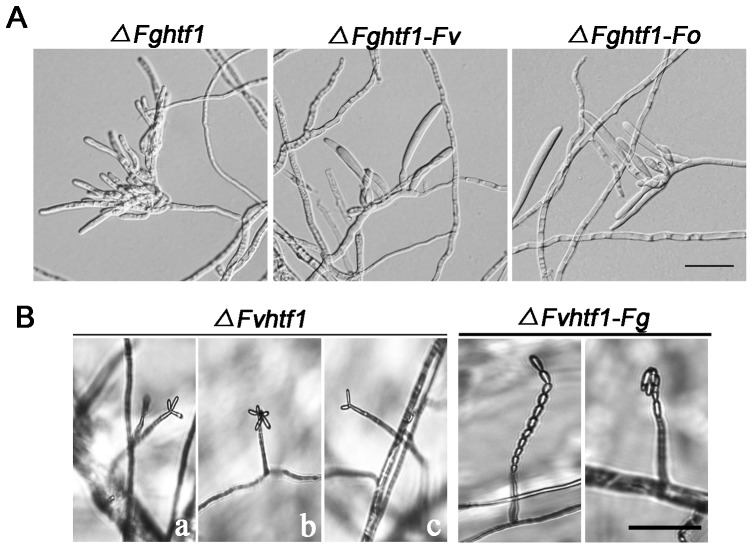
Complementation of Δ*Fghtf1* and Δ*Fvhtf1* mutant by *Fusarium* Htf1 homologous genes. (A) We complemented *F. graminearum* gene deletion mutant (Δ*Fghtf1*) with *F. verticillioides FvHTF1* (Δ*Fghtf1-Fv*) and *F. oxysporum FoHTF1* (Δ*Fghtf1-Fo*). Δ*Fght1-Fv* and Δ*Fght1-Fo* produced abundant macroconidia borne on terminal phialides in contrast to Δ*Fghtf1* which produced aberrant terminal phialides and failed to form macroconidia and. Bar = 20 µm. (B) In *F. verticillioides* gene deletion mutant (Δ*Fvht1*), microconidial chains are absent instead microconidia assemble in abnormal false heads (a–c). *F. graminearum FgHTF1* complemented strain (Δ*Fvht1-Fg*) showed protypical chains and false heads of microconidia found in wild-type *F. verticillioides*. Bar = 50 µm.

Htf1 in three *Fusarium* species showed highly conserved functions in macroconidiogenesis and microconidiogenesis. *F. graminearum* FgHtf1 homeodomain and whole protein sequence share greater than 95% and 85% amino acid identity, respectively, when compared to FvHtf1 and FoHtf1 (Figure 1B). To test whether Htf1 homologs from three *Fusarium* species are functional orthologs, we transformed *FvHTF1* and *FoHTF1* genes with their respective promoter regions into *ΔFghtf1* mutant. Positive transformants were identified by PCR with respective specific primers (Table S2), and these showed rescued conidiogenesis in *ΔFghtf1* mutant when incubated in CMC medium (Figure 9A, Table 1). In addition, we also transformed *FgHTF1* gene into the *ΔFvhtf1* mutant. Significantly, the complemented strain *ΔFvhtf1-Fg* produced abundant microconidia in chain and false head shape although *F. graminearum* species does not produce microconidia (Figure 9B). These results suggest that Htf1 is conserved in three Fusaria and that FgHtf1 can transcriptionally regulate *F. verticillioides* proteins that are involved in microconidiogenesis.

## Discussion

**Figure 10:**
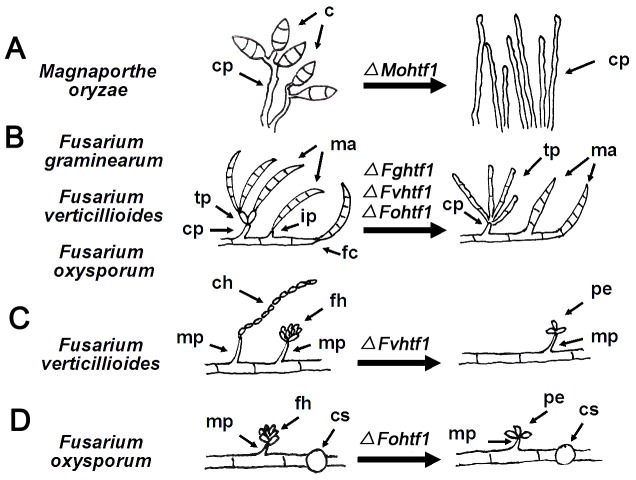
Proposed role of Htf1 in *M. oryzae*, *F. graminearum*, *F. verticillioides* and *F. oxysporum*. (A) MoHtf1 is important for proper conidiophore development and subsequent conidia formation. (B) In three Fusarium species, Htf1 plays a critical role in proper development of phialides. *HTF1* gene deletion led to aberrant terminal phialide and ultimately abolished macroconidia formation from conidiophores. Macroconidia formation directly from hyphae were also affected, namely in foot cell development. (C) In *F. verticillioides ΔFvhtf1* mutants, prototypical microconidia chain and false heads were not observed but rather a 2- to 5- microconidia complex at the apex of monophialides resulting in a floral petal-like shape. This suggests that FvHtf1 is important for basipetal division typically observed in the wild-type *F. verticillioides.* (D) In *F. oxsporum ΔFohtf1* mutants, we observed floral petal-shaped microconidia false head similar to *F. verticillioides ΔFvhtf1* mutants suggesting FoHtf1 is important for phialide development and subsequent microconidiogenesis. However, it is not involved in chlamydospore development. c, conidia; cp, conidiophore; ma, macroconidia; tp, terminal phialide; ip, intercalary phialide; fc, foot cell; ch, chain; mp, monophialide; fh, false head; pe, petal; cs, chlamydospore.

Conidiation is an important characteristic in fungi that requires spatial and temporal regulation of gene expression that leads to specialized cellular differentiation and intercellular communications [1], [2], [10]. In our previous study, we found that *HTF1* is essential for conidiation in *M. oryzae*. Further observation revealed that *ΔMohtf1* mutant produces greater amounts of conidiophores, which showed curvature slightly near the tip but could not develop into sterigmata-like structures (Figure 10) [50]. This led us to conclude that *MoHTF1* is an essential positive regulator responsible for switching from conidiophore maturation to the initiation of conidia development in *M. oryzae*. Concurrently, we also proposed that *MoHTF1* functions as a negative regulator of conidiophore development. In other filamentous fungi, homeodomain transcription factors have been linked to the shaping of fruiting body structure, sexual reproduction, and mycelial branch formation [49], [54]–[56]. In this study, we hypothesized that, while there are similarities and conservation in *HTF1* gene function between *M. oryzae* and *Fusarium* species, there are divergent biological features exhibited by Htf1 in *Fusarium* species. In three *Fusarium* species, we found that the deletion of *HTF1* also abolished macroconidia development from conidophores. In addition, *Δhtf1* mutants in these *Fusarium* species failed to form morphologically discernible phialide, but rather forming “clusters” consisting of hyphal segments (Figure 10). This phenotype was more profound in *F. graminearum* than *F. verticillioides* and *F. oxysporum*, however it was consistently observed in three Fusaria. In addition, the deletion of *HTF1* in *Fusarium* species caused excessive elongation of conidiogenous cell, suggesting that Htf1 is a negative regulator of conidiogenous cell development similar to *M. oryzae*.

But we also discovered a major difference in conidiogenesis between *M. oryzae* and *Fusarium* species. In *M. oryzae*, the conidiogenous cell usually was deemed as conidiophore, while in *Fusarium* species it often represent phialides, and therefore it would be reasonable to presume that the function of *HTF1* in *Fusarium* species underwent a further specialization for phialidegenesis. Some genes associated with phialide development have been reported in *Fusarium* species, such as *FgStuA* in *F. graminearum*, *FoStuA* and *REN1* in *F. oxysporum.* The sequence of FgStuA protein showed a very high level of homology (72%) with FoStuA [27], [29]. Not surprisingly, the deletion mutants of *ΔFgStuA* and *ΔFoStuA* lacked conidiophores and uninucleate phialides, suggesting a conserved function in two orthologs. In *A. nidulans*, where phialidic conidiation has been extensively studied, *stuA* mutants produced significantly stunted conidiophores and lacked normal metulae and phialides [57], [58]. It is also recognized that *stuA* affects conidiation through the spatial and temporal modifier of *brlA* and *abaA* expression [59], [60]. However, FgStuA regulates sexual development and pathogenicity in addition to conidiogenesis, suggesting that this transcription factor may have a broader and diverse impact on *F. graminearum* lifestyle [27]. Significantly, in contrast to FgStuA all gene-deletion mutants we studied (*ΔMohtf1*, *ΔFghtf1*, *ΔFvhtf1*, *ΔFohtf1*) showed phenotypic deformity only limited to conidiogenesis. Our SAGE data (unpublished) and previously published microarray study in PH-1 and *ΔFgStuA* [27] showed no reciprocal influence in *FgHTF1* and *FgSTUA*, and it is reasonable to hypothesize that FgStuA and FgHtf1 regulate phialide development through different cellular networks.

*REN1* encodes a protein analogous to *A. nidulans* MedA and *M. oryzae* Acr1, and all of these are involved in conidiogenesis [28]. The *ren1* mutant strains lacked normal conidiophores and phialides and formed rod-shaped, conidium-like cells directly from hyphae by acropetal division [28], but maintained pathogenicity on host. These results showed that Ren1 specifically regulate conidiogenesis. In our study, we concluded that *FgHTF1* does not directly play a role in conidial germination and pathogenicity, although there were many significant defects in phialidiogenesis and macroconidiogenesis in *ΔFghtf1* mutant. This similarity in cellular function led us to further analyze the expression of *REN1* orthologous gene FGSG_02471 in *ΔFghtf1* mutant. However, the result showed no significant change in expression (data not shown), suggesting that these two transcription factors are not epistatic. However, it remains to be tested whether these two genes regulate signaling pathways that converge downstream and impact conidiogenesis in *F. graminearum*.

In *Fusarium* species, macroconidia have distinct basal foot cell and pointed distal ends. In this study, we discovered that macroconidia in *Δhtf1* mutants, those produced through hyphal budding, exhibit significant defect in the foot cell (Figure 10). When we monitored conidiogenesis in microscopic detail, we recognized that the first initial conidium of the wild-type strain is formed within the apical extension of the phialide or hyphae at the early stage of development. Before the macroconidium is released, the characteristic foot cell at the base of macroconidium is formed. Nevertheless, at late stages of development, the expanding conidium ruptures the conidiophore wall and is released by an abscissional splitting of the basal septum. Internal septation of the conidium normally occurs after it is released. In the *Δhtf1* mutant, it is unlikely that a conidium is released by abscissional splitting of the basal septum (Figure 10). Perhaps this is why we found mature conidium with distinct septum born on hyphae in the *Δhtf1* mutant. This implies that Htf1 play a key role in regulating foot cell division during macroconidiogenesis.

Production of both microconidia and macrocondia is a common phenomenon in most *Fusarium species*, and both conidia are formed from phialides in false heads by basipetal division, the developmental mode from the apex toward the base without catenation of cells [28]. In our study, we learned that Htf1 not only regulated macroconidiogenesis, but also for microconidiogenesis. In *ΔFvhtf1* mutant, long chains of microconidia were completely absent in contrast to the wild-type strain (Figure 10). In *F. verticillioides*, the cAMP signaling pathway gene *FAC1* and two hydrophobin genes *HYD1* and *HYD2* have been reported to be important for the production of microconidial chains [39]. In addition, Choi and Xu [36] further showed that *FAC1* positively regulates microconidia production and the expression of two hydrophobin genes, *HYD1* and *HYD2* [36]. Notably, all these reported genes had no discernable effect on false-head pattern of microconidia, suggesting that these gene-deletion mutants still can produce microconidia through basipetal division. Intriguingly, our study revealed that the deletion of *HTF1* in *F. verticillioides* and *F. oxysporum* led to the formation of petal-shaped pattern by sharing the apical branches (Figures 6 and 10) instead of producing typical false heads with characteristic ball-shaped assemblage of microconidia held together apparently by mucilage. As we described earlier, this aberrant microconidiogenesis from monophialide may have interfered with typical microconidia chain development that occurs through basipetal division and ultimately led to significant reduction in microconidia in *F. verticilioides* and *F. oxysporum*. Therefore, our results collectively provide evidences that Htf1 regulates microconidial formation from chains and false heads by basipetal division in *Fusarium* species.

## Materials and Methods

### Strains, media and growth condition

All wild-type and mutant strains used in this study are listed in Table S1. In *F. graminearum*, growth and morphology were evaluated by culturing strains on complete medium (CM: 0.6% yeast extract [w/v], 0.6% casein hydrolysate [w/v], and 1% sucrose [w/v]) at 28°C for 4 days. Formation of perithecia was assayed on wheat kernels medium as described previously [61]. To assay conidiation, an agar block (3 mm in diameter) carrying mycelia was introduced into 50 ml of liquid CMC medium [52]. The suspension was shaken at 180 rpm for 3–15 days, and the concentration of conidia was determined with a hemacytometer. For spore germination assays, fresh macroconidia were suspended in CM for 4 h with gentle agitation [62]. Macroconidia of PH-1 and mutants were observed using an Olympus BX51 Microscope. Infection assays on flowering wheat heads, wheat coleoptiles and corn stalks were conducted as previously described [63]–[65].

*F. verticillioides* strains were grown on CM agar and mung bean agar medium (5% mung bean [w/v], 1.5% agar [w/v], pH 6.0) to observe morphology and growth. For macro- and microconidiation assays, a culture block (3 mm in diameter) was inoculated on synthetic low-nutrient agar (SNA) medium, containing (all in w/v) 0.1% KH_2_PO4, 0.1% KNO_3_, 0.05% MgSO_4_ ·7H_2_O, 0.05% KCl, 0.02% glucose, 0.02% sucrose, and 2% agar, and mung bean agar or broth. After incubation at 25°C for 7 days under continuous near-ultraviolet (UV) light or dark condition [66], conidiation was observed under a light microscope (Olympus BX51).

The *F. oxysporum* wild-type and mutant strains were cultured on CM agar to observe morphology and growth. To induce conidiation in *F. oxysporum* strains, SNA and mung bean agar were used as described above. All tests were repeated three times.

### Microscopy and histological visualization

To observe conidiogenesis in *F. graminearum*, an agar block carrying mycelium was inoculated into CMC as described above and then were imaged at different culture stages with Olympus BX51 Research Microscope. Nuclear visualization in phialides was observed by DAPI staining. Mycelia were collected by centrifugation, washed with PBS buffer (pH 7.2) and then resuspended in PBS containing 0.1% Triton X-100. Cells were then fixed with PBS paraformaldehyde (3.7%, w/v) and stained with 10 µg/ml DAPI (Sigma). The cell nuclei were observed with Olympus BX51 Research Microscope at UV excitation wavelength. For spore germination studies, fresh PH-1 macroconidia were suspended in CM for 4 h with gentle agitation. Cell walls and septa of germinating conidia were visualized by staining with Calcofluor White (10 mg/ml, Sigma).

To directly visualize *F. verticillioides* and *F. oxysporum* conidial chains and false heads without immersion in water or buffer, agar squares were removed from actively growing colonies and placed in a slide glass with the fungal colony surface oriented perpendicular to the cover slip. Images were acquired from Olympus BX51 Research Microscope.

For scanning electron microscopy (SEM), blocks of 5-day-old mung bean agar cultures (5 mm^2^) were fixed in 4% glutaraldehyde at 4°C for 16 h. The samples were then dehydrated in a graded ethanol series and dried in a critical point dryer as described [67]. Samples were coated with a thin gold layer and observed with JSM-6360LV (Jeol Ltd., Tokyo) scanning electron microscope.

### Fungal transformation and generation of gene-deletion mutants

The *F. graminearum*, *F. verticillioide* and *F. oxysporum* protoplast preparation and fungal transformation were performed following standard protocols [29], [53], [63]. Hygromycin- or neomycin-resistant transformants were selected on media supplemented with 250 g/mL hygromycin B (Roche Applied Science) or 200 g/mL G418 (Invitrogen).

To generate the *ΔFghtf1* mutant, a 1,291-bp fragment upstream from *FgHTF1* was amplified with primers FG07097AF and FG07097AR, and this amplicon was subsequently cloned into the *Hin*dIII and *Eco*RI sites upstream of the *hph* cassette on pCX63 [68]. Then, 1,033-bp fragment downstream from *FgHTF1* was amplified with primers FG07097BF and FG07097BR, and cloned into the *Bam*HI and *Sac*I sites downstream of *hph* cassette, and this plasmid was transformed into protoplasts of the wild-type PH-1 strain as described [63]. Hygromycin-resistant transformants were screened by PCR with primers FG07097UA and H853 and primers FG07097OF and FG07097OR (Table S2). An isolate that tested positive with PCR was further verified by Southern blot analysis performed with the digoxigenin high prime DNA labeling and detection starter Kit I (Roche, Mannheim, Germany).

We generated *HTF1* gene-replacement constructs in *F. verticillioide* and *F. oxysporum* using the split-marker approach [69], [70]. Upstream and downstream fragments were amplified with specific primer pairs that are listed in Table S2. Partial fragments of the hygromycin phosphotransferase (*hph*) gene were amplified with primers HYG/F, HY/R, YG/F, and HYG/R as described [71]. After transformation, hygromycin-resistant transformants were screened by PCR with designated primers (Table S2) and further characterized by Southern blot analysis.

For complementation of *ΔFghtf1* and *ΔFvhtf1*, *FgHTF1* and *FvHTF1* (with upstream promoter and downstream terminator) was amplified with primer sets FG07097CF4/FG07097CR4 and FV08072CF/FV08072CR, respectively (Table S2). The resulting constructs were co-transformed into protoplasts of the target mutant along with a vector harboring geneticin-resistance marker (pKNTG). Transformants exhibiting resistance to both geneticin and hygromycin were selected, screened by PCR for the presence of the complementation construct, and further validated by Southern blot analyses. Inter-species complemented strategies were similar to generation of *ΔFghtf1-Com* strain. *FgHTF1*, *FvHTF1* or *FoHTF1* gene was amplified with a set of primers (Table S2), and subsequently co-transformed into the target fungal protoplasts with pKNTG vector. The selected isolates were further analyzed by PCR, using primers (Table S2) to determine the presence of *FgHTF1*, *FvHTF1* or *FoHTF1* gene.

### Construction of *FgHTF1-GFP* vector and complementation

The FgHTF1-GFP fusion vector, pGM-FgHTF1-GFP, was constructed by amplification of 2,886-bp fragment including 1,459-bp *FgHTF1* coding sequence and a 1,427-bp promoter region using primers FG07097CF3-GFP and FG07097CR3-GFP (Table S2). The 2,886-bp PCR product was then cloned into pGEM-T easy vector to generate pGM-FgHTF1. The 1.5-kb GFP allele [72] carrying the *A. nidulans trpC* terminator was amplified using primers HindIII-GFPF and HindIII-GFPR (Table S2), then cloned into pGEM-T easy vector. It was subsequently digested with *Hin*dIII to release the GFP allele with *Hin*dIII sticky ends, which was inserted into *Hin*dIII site of pGM-FgHTF1 to create pGM-FgHTF1-GFP. We verified the orientation of GFP insertion and in-frame fusion by sequencing the pGM-FgHTF1-GFP vector. To generate FgHTF1-GFP strain, pGM-FgHTF1-GFP vector and pKNTG vector were cotransformed into *ΔFghtf1* mutant. Transformants carrying a single insertion were selected and phenotypic restoration in *ΔFghtf1* mutants was sought. GFP fluorescence was observed using a Leica TCS SP5 inverted confocal laser scanning microscope (Leica, Germany)

### Quantitative RT–PCR

Wild-type conidia were harvested at growth stages (24 h, 36 h, 48 h, 60 h and 72 h incubated on CMC medium). RNA was isolated with TRIzol reagent (Invitrogen) and purified with the DNA-free kit (Ambion). First-strand cDNA was synthesized with the M-MLV reverse transcriptase (Invitrogen), and qRT-PCR was performed with the ABI 7500 sequence detection system (Applied Biosystem) using QuantiTect SYBRgreen PCR Master Mix (Qiagen). Primers used to amplify selected genes in qRT-PCR reactions are listed in supplemental Table S2. *TUB2* (FGSG_06610.3) was used as the endogenous reference gene. The relative quantification of each transcript was calculated by the 2^−*ΔΔ*C^_T_ method [73]. All qRT-PCR reactions were conducted in triplicates for each sample and the experiment was repeated three times.

## Supporting Information

Table S1 **Wild-type and mutant strains of fungi used in this study.**
(DOC)

Table S2 **PCR primers used in this study.**
(DOC)

Figure S1 **Analysis of putative Htf1 homeobox transcription factors in fungi.** (A) Schematic description of *HTF1* gene structure, namely intro/exon boundaries, in *Fusarium* species (*FgHTF1*, *FvHTF1*, and *FoHTF1*) and *Magnaporthe oryzae* (*MoHTF1*). Gray blocks and gray lines indicate exons and introns, respectively. Numbers on right indicate deduced protein sequence length in amino acids. (B) Sequence alignment of *F. graminearum* (FgHtf1), *F. verticillioides* (FvHtf1), *F. oxysporum* (FoHft1) and *M. oryzae* (MoHtf1) predicted protein sequences was performed using Clustal W and Boxshade (http://bioweb.pasteur.fr/seqanal/interfaces/boxshade.html). The conserved amino acid residues are shaded black, whereas similar residues are shown in gray. Consensus amino acids are marked with asterisk (*).
(TIFF)

Figure S2 **The** ***FgHTF1*** **gene-replacement construct and mutants.** (A) Schematic diagram of the genomic region of the *FgHTF1* and *hph* genes. Primers F1 (FG07097AF), R1 (FG07097AR), F2 (FG07097BF) and R2 (FG07097BR) were used to generate *FgHTF1* gene replacement constructs, and OF1 (FG07097OF), OR1 (FG07097OR), F1 (FG07097AF) and R1 (FG07097AR) were used for mutant screening and identification. S, *Sal* I. (B) DNA gel blots of *Sal* I-digested genomic DNA were hybridized with *FgHTF1* upstream fragment as the probe (shown in Figure S2A). PH-1, wild-type strain; *ΔFghtf1-Com*, complementation strain; *ΔFghtf1-7* and *ΔFghtf1-8*, null mutants. (C) Total RNA samples isolated from mycelia of PH-1, *ΔFghtf1-8* and *ΔFghtf1-Com* were subjected to RT-PCR using *FgHtf1* gene-specific primers FG07097OF and FG07097OR (Table S3). As predicted, the RT-PCR amplicon (1,178 bp) was observed in PH-1 and *ΔFghtf1-Com*, but was absent the deletion mutant *ΔFghtf1-8*.
(TIFF)

Figure S3 **Vegetative growth and fertility in** ***F. graminearum*** **wild type (PH-1) and** ***ΔFghtf1*** **mutant.** (A) Colonies PH-1 and *ΔFghtf1* grown on CM agar for 4 days. (B) PH-1 and *ΔFghtf1* were incubated on wheat kernels medium for 2 weeks to induce formation of perithecia. No significant difference was observed.
(TIFF)

Figure S4 ***F. verticillioides FvHTF1*** **gene-replacement strategy and confirmation.** (A) Schematic diagram of the genomic region of the *FvHTF1* and *hph* genes. Primers F1 (FV08072AF), R1 (FV08072AR), F2 (FV08072BF) and R2 (FV08072BR) were used to generate *FvHTF1* gene replacement constructs. Probe 1 and probe 2 were used to screen and verify gene replacement mutants. K, *Kpn* I. (B) DNA gel blots of *Kpn*I-digested genomic DNA were hybridized with probe 1 and probe 2. A149, wild-type *F. verticillioides*; *ΔFvhtf1-Com*, complementation strain; *ΔFvhtf1-9* and *ΔFvhtf1-15*, null mutants.
(TIFF)

Figure S5 **Colony and microconidia morphology of** ***F. verticillioides*** **wild type (A149) and** ***ΔFvhtf1*** **mutant.** (A) Colony morphology of A149 and *ΔFvhtf1* mutant grown on CM and MB agar for 6 and 7 days, respectively. (B) Microconidia stained with 4′6-diamidino-2-phenylindole (DAPI) observed under a fluorescence microscope. Bar = 10 µm.
(TIFF)

Figure S6 ***F. oxysporum FoHTF1*** **gene-replacement strategy and confirmation.** (A) Schematic diagram of the genomic region of the *FoHTF1* and *hph* genes. Primers F1 (FO01706AF), R1 (FO01706AR), F2 (FO01706BF) and R2 (FO01706BR) were used to generate *FoHTF1* gene replacement constructs. Probe was used to screen and verify gene replacement mutants. N, *Nco*I. (B) DNA gel blots of *Nco*I-digested genomic DNA were hybridized with probe. WT, wild-type *F. oxysporum*. *ΔFohtf1-3*, *ΔFohtf1-6*, *ΔFohtf1-9* and *ΔFohtf1-11*, null mutants. *ΔFohtf1-Ect*, ectopic strain.
(TIFF)

Figure S7 **Colony and microconidia morphology of** ***F. oxysporum*** **wild type (WT) and** ***ΔFohtf1*** **mutant.** (A) Colony morphology of WT and *ΔFohtf1* mutant grown on CM agar for 6 days. (B) Microconidia stained with 4′6-diamidino-2-phenylindole (DAPI) observed under a fluorescence microscope. Bar = 10 µm.
(TIFF)
