# Supplementary material for: A Conserved Homeobox Transcription Factor Htf1 Is Required for Phialide Development and Conidiogenesis in Fusarium Species
Source: PLoS One. 2012 Sep 21;7(9):e45432. doi: 10.1371/journal.pone.0045432 (PMC3448628; doi:10.1371/journal.pone.0045432)
Supplement: Table S1 — Wild-type and mutant strains of fungi used in this study. (DOC) [file pone.0045432.s001.doc]

**Table S1**. **Wild-type and mutant strains of fungi used in this study**

| **Strain** | **Genotype description** | **phenotype** | **Source** |
| --- | --- | --- | --- |
| PH-1 | Wild-type | Control | FGSC* |
| *ΔFghtf1* | FGSG_07097.3 deletion mutant in PH-1 background | Conidiation and phialides cell development | This study |
| *ΔFghtf1-Com/ΔFghtf1-GFP* | *ΔFghtf1* transformant expressing the FgHtf1 or FgHtf1-GFP construct | Recover the conidiation of *ΔFghtf1* mutant | This study |
| A149 | Wild-type | Control | FGSC* |
| *ΔFvhtf1* | FVEG_08072.3 deletion mutant in A149 background | Conidiation and phialides cell development | This study |
| *ΔFvhtf1-Com* | *ΔFvhtf1* transformant expressing the FvHtf1 construct | Recover the conidiation of *ΔFvhtf1* mutant | This study |
| FOL 4287 | Wild-type | Control | FGSC* |
| *ΔFohtf1* | FOXG_01706.2 deletion mutant in FOL 4287 background | Conidiation and phialides cell development | This study |
| *ΔFghtf1-Fv* | *ΔFghtf1* transformant expressing the FvHtf1 construct | Recover the conidiation of *ΔFghtf1* mutant | This study |
| *ΔFghtf1-Fo* | *ΔFghtf1* transformant expressing the FoHtf1 construct | Recover the conidiation of *ΔFghtf1* mutant | This study |
| *ΔFvhtf1-Fg* | *ΔFvhtf1* transformant expressing the FgHtf1 construct | Recover the conidiation of *ΔFvhtf1* mutant | This study |

* FGSC: Fungal Genetic Stock Center. Kansas City, Kansas, USA
