# Supplementary material for: A Conserved Homeobox Transcription Factor Htf1 Is Required for Phialide Development and Conidiogenesis in Fusarium Species
Source: PLoS One. 2012 Sep 21;7(9):e45432. doi: 10.1371/journal.pone.0045432 (PMC3448628; doi:10.1371/journal.pone.0045432)
Supplement: Table S2 — PCR primers used in this study. (DOC) [file pone.0045432.s002.doc]

**Table S2. PCR primers used in this study**

| Primers | Sequence(5’-3’) | Application |
| --- | --- | --- |
| FG07097AF | CCCAAGCTTAGACTTGCTGTTGACTCCCACT | *FgHTF1* deletion and probe |
| FG07097AR | CCGGAATTCGCCGCTTCTGAGGAATGTAA |  |
| FG07097BF | CGGGATCC ATGGGAATACGGCACTGGA |  |
| FG07097BR | CGGAGCTC CATGCTGGAAGAGGAAACG |  |
| FG07097OF | CCCAAGTCAAAACAGGTCT | *∆Fghtf1* mutant screen |
| FG07097OR | CGAAGCCAAAATAGGTAGG |  |
| FG07097UA | TATCCAATCCAATGCCTTCA | *∆Fghtf1* mutant screen |
| H853 | GACAGACGTCGCGGTGAGTT |  |
| FG07097CF3-GFP | AACGGCGAAAGGGTAATAT | *∆Fghtf1* complementation and GFP fusion |
| FG07097CR3-GFP | CCCAAGCTT CTTGTCTGAGTAGGTGAAGCTC |  |
| Hind III -GFPF | CCCAAGCTT ATGGTGAGCAAGGGCGAGGAGC | GFP fusion |
| Hind III -GFPR | CCCAAGCTT GTGGAGATGTGGAGTGGGCGCTT |  |
| FG07097CF4 | AGGGCGAAGATAGATACCAGAC | *∆Fghtf1* and *∆Fvhtf1* complementation |
| FG07097CR4 | GGGACACCACCTCAAAGATG |  |
| FG07097QF | CGCTCGCATCAACAACTG | qRT-PCR |
| FG07097QR | CGTCGTCTGAATGAACACC |  |
| TUB2F | GTCAGTGCGGTAACCAAATCGGT | qRT-PCR |
| TUB2R | CTCAGAGGTGCCGTTGTAAACACC |  |
| FV08072AF | TTGTCGTTGGGAAGGGTG | *FvHTF1* deletion |
| FV08072AR | TTGACCTCCACTAGCTCCAGCCAAGCCTGCTGTTGGGCTTGTGATT |  |
| FV08072BF | GAATAGAGTAGATGCCGACCGCGGGTTCCTTCCTACTTTGGCTTCG |  |
| FV08072BR | TCGGGTTTGGACTAATGTGA |  |
| HYG/F | GGCTTGGCTGGAGCTAGTGGAGGTCAA | Gene deletion and probe |
| HY/R | GTATTGACCGATTCCTTGCGGTCCGAA |  |
| YG/F | GATGTAGGAGGGCGTGGATATGTCCT |  |
| HYG/R | AACCCGCGGTCGGCATCTACTCTATTC |  |
| FV08072OF | GGCACTGGCTGAGTCTATGAG | *∆Fvhtf1* screen and probe |
| FV08072OR | CTGAGTAACTGCGGGAGGC |  |
| FV08072UA | GCGCACGCCTAAAACAAA | *∆Fvhtf1* screen |
| FV08072CF | ACTGACGGCTCCAACACCTAC | *∆Fvhtf1* and *∆Fghtf1*  complementation |
| FV08072CR | CAAACCTTTCCCTCGCAACA |  |
| FO01706AF | TGCGTCTCACAGAGTCAATCA | *FoHTF1* deletion |
| FO01706AR | TTGACCTCCACTAGCTCCAGCCAAGCCCGTTAGGTAGCGGAACAAGTC |  |
| FO01706BF | GAATAGAGTAGATGCCGACCGCGGGTTACCGCCCACTCCCAATACA |  |
| FO01706BR | CCTCCGTGATGATAATAAACTCC |  |
| FO01706OF | CACAAGCCCAACAGCACTAC | *∆Fohtf1* screen |
| FO01706OR | AGACTGGTCCACAGCAAGGT |  |
| FO01706UA | CTGCCTCATCTCACCTCCCA | *∆Fohtf1* screen |
| FO01706CF | CTGCCTGCCTTATGGATACTTT | *∆Fghtf1*  complementation |
| FO01706CR | CGGGTTTGGGCTAATGTGA |  |
