# Supplementary figures and images for: A Conserved Homeobox Transcription Factor Htf1 Is Required for Phialide Development and Conidiogenesis in Fusarium Species
Source: PLoS One. 2012 Sep 21;7(9):e45432. doi: 10.1371/journal.pone.0045432 (PMC3448628; doi:10.1371/journal.pone.0045432)

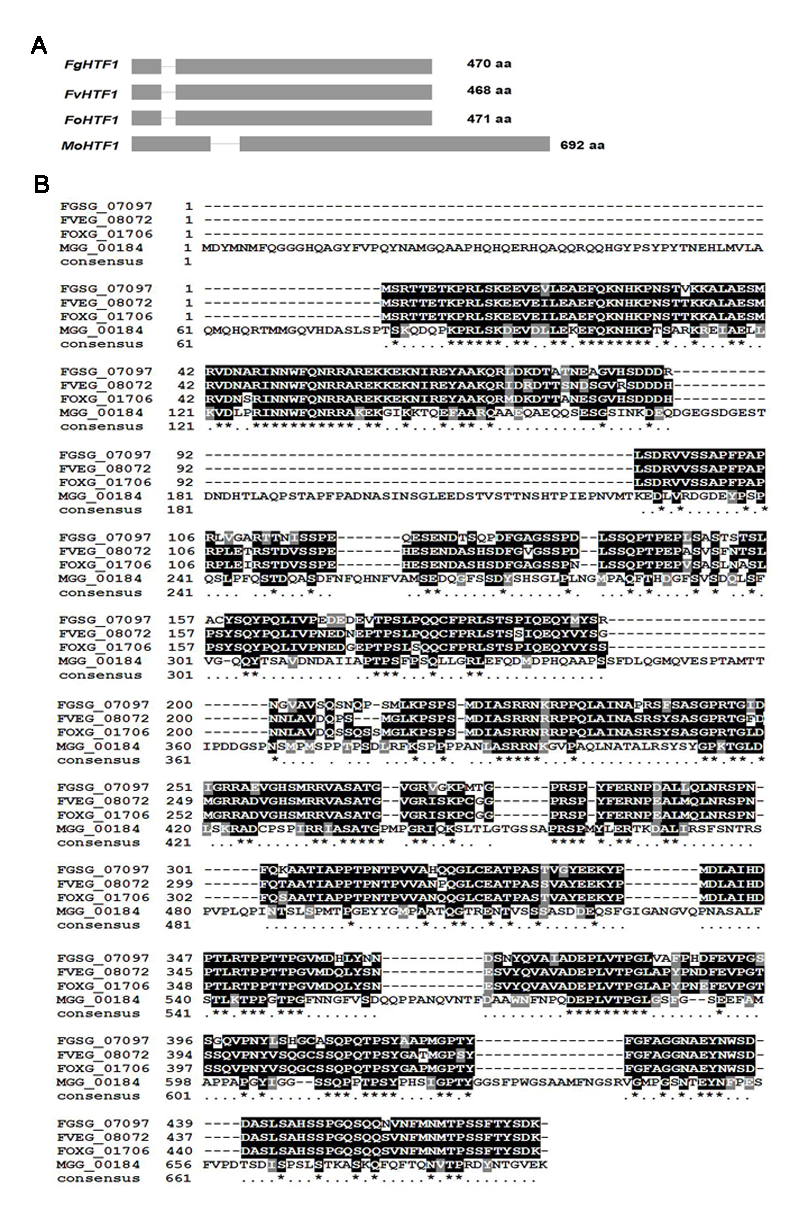

Supplement: Figure S1 — Analysis of putative Htf1 homeobox transcription factors in fungi. (A) Schematic description of HTF1 gene structure, namely intro/exon boundaries, in Fusarium species (FgHTF1, FvHTF1, and FoHTF1) and Magnaporthe oryzae (MoHTF1). Gray blocks and gray lines indicate exons and introns, respectively. Numbers on right indicate deduced protein sequence length in amino acids. (B) Sequence alignment of F. graminearum (FgHtf1), F. verticillioides (FvHtf1), F. oxysporum (FoHft1) and M. oryzae (MoHtf1) predicted protein sequences was performed using Clustal W and Boxshade (http://bioweb.pasteur.fr/seqanal/interfaces/boxshade.html). The conserved amino acid residues are shaded black, whereas similar residues are shown in gray. Consensus amino acids are marked with asterisk (*). (TIFF) [file pone.0045432.s003.tiff]

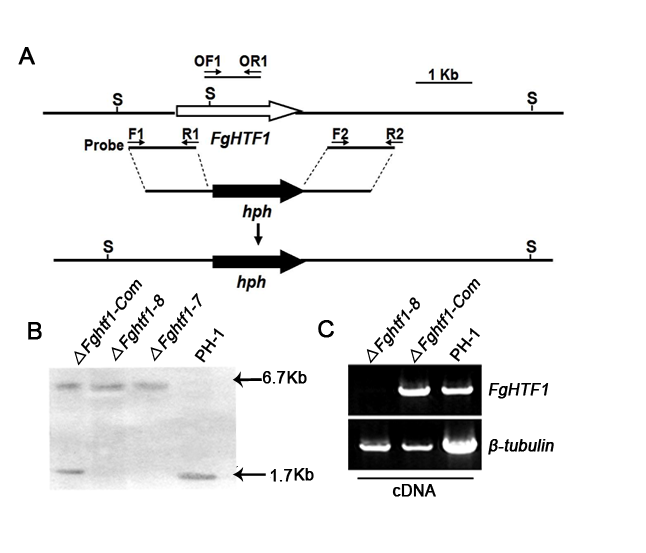

Supplement: Figure S2 — The FgHTF1 gene-replacement construct and mutants. (A) Schematic diagram of the genomic region of the FgHTF1 and hph genes. Primers F1 (FG07097AF), R1 (FG07097AR), F2 (FG07097BF) and R2 (FG07097BR) were used to generate FgHTF1 gene replacement constructs, and OF1 (FG07097OF), OR1 (FG07097OR), F1 (FG07097AF) and R1 (FG07097AR) were used for mutant screening and identification. S, Sal I. (B) DNA gel blots of Sal I-digested genomic DNA were hybridized with FgHTF1 upstream fragment as the probe (shown in Figure S2A). PH-1, wild-type strain; ΔFghtf1-Com, complementation strain; ΔFghtf1-7 and ΔFghtf1-8, null mutants. (C) Total RNA samples isolated from mycelia of PH-1, ΔFghtf1-8 and ΔFghtf1-Com were subjected to RT-PCR using FgHtf1 gene-specific primers FG07097OF and FG07097OR (Table S3). As predicted, the RT-PCR amplicon (1,178 bp) was observed in PH-1 and ΔFghtf1-Com, but was absent the deletion mutant ΔFghtf1-8. (TIFF) [file pone.0045432.s004.tiff]

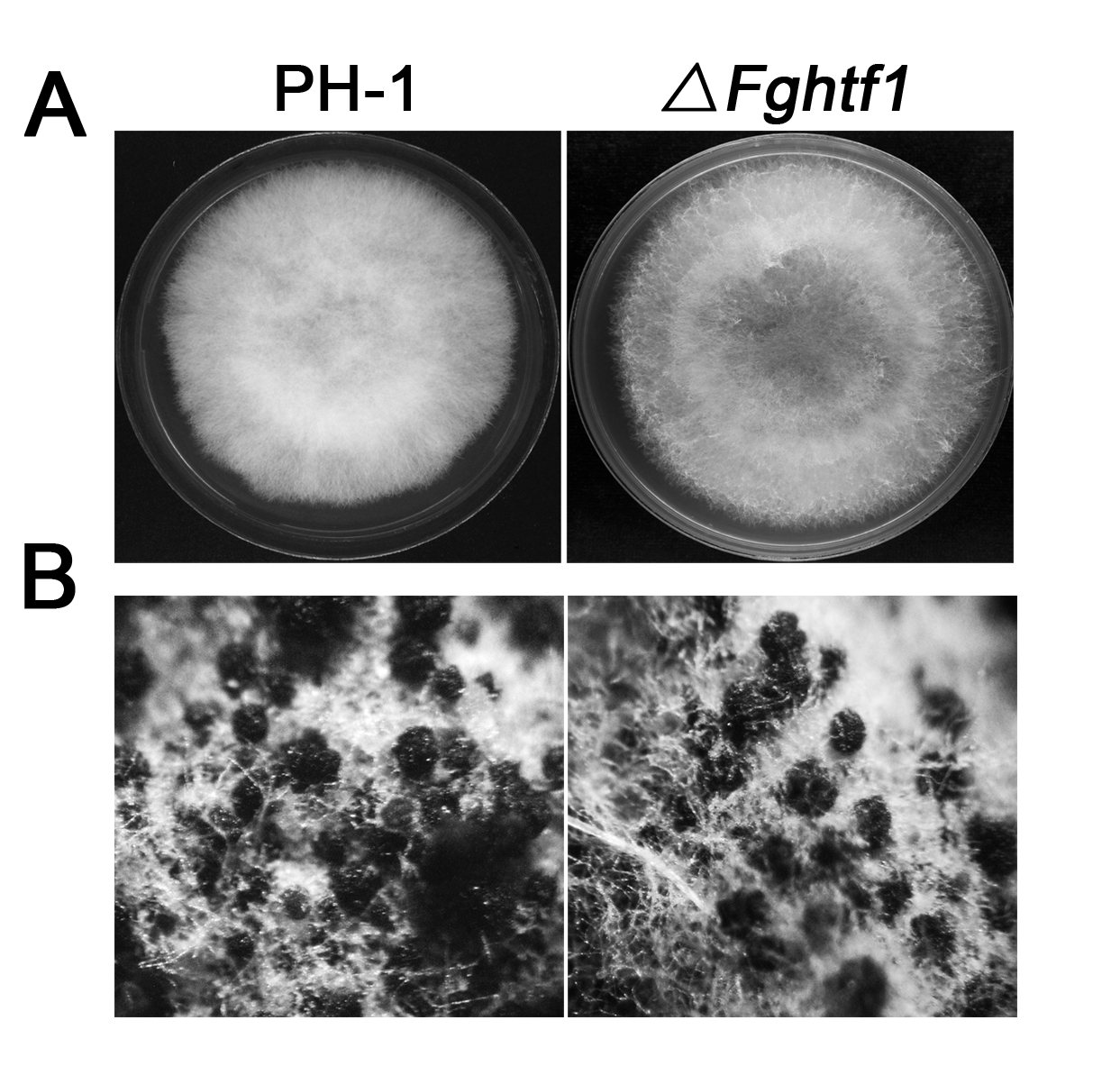

Supplement: Figure S3 — Vegetative growth and fertility in F. graminearum wild type (PH-1) and ΔFghtf1 mutant. (A) Colonies PH-1 and ΔFghtf1 grown on CM agar for 4 days. (B) PH-1 and ΔFghtf1 were incubated on wheat kernels medium for 2 weeks to induce formation of perithecia. No significant difference was observed. (TIFF) [file pone.0045432.s005.tiff]

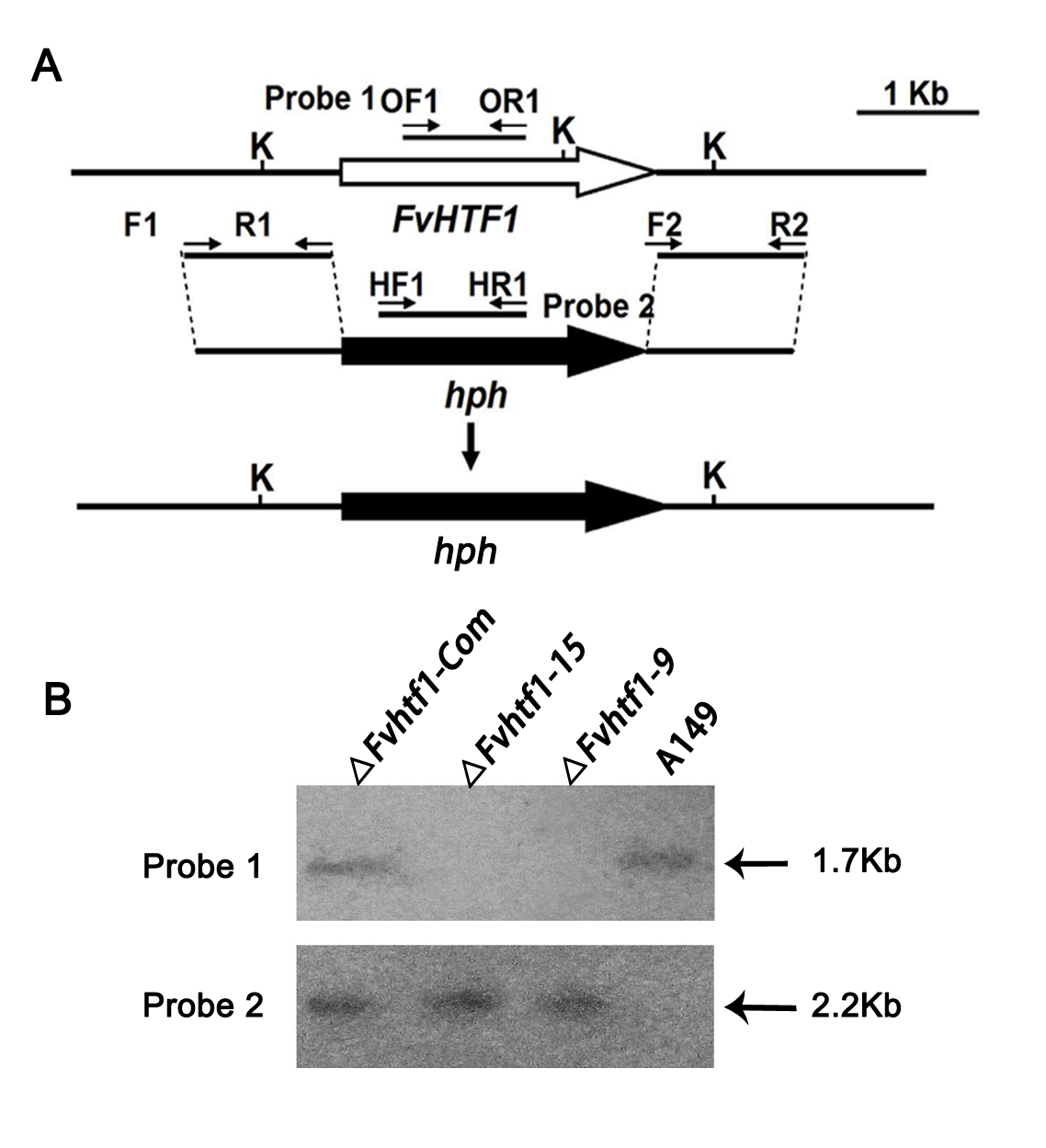

Supplement: Figure S4 — F. verticillioides FvHTF1 gene-replacement strategy and confirmation. (A) Schematic diagram of the genomic region of the FvHTF1 and hph genes. Primers F1 (FV08072AF), R1 (FV08072AR), F2 (FV08072BF) and R2 (FV08072BR) were used to generate FvHTF1 gene replacement constructs. Probe 1 and probe 2 were used to screen and verify gene replacement mutants. K, Kpn I. (B) DNA gel blots of KpnI-digested genomic DNA were hybridized with probe 1 and probe 2. A149, wild-type F. verticillioides; ΔFvhtf1-Com, complementation strain; ΔFvhtf1-9 and ΔFvhtf1-15, null mutants. (TIFF) [file pone.0045432.s006.tiff]

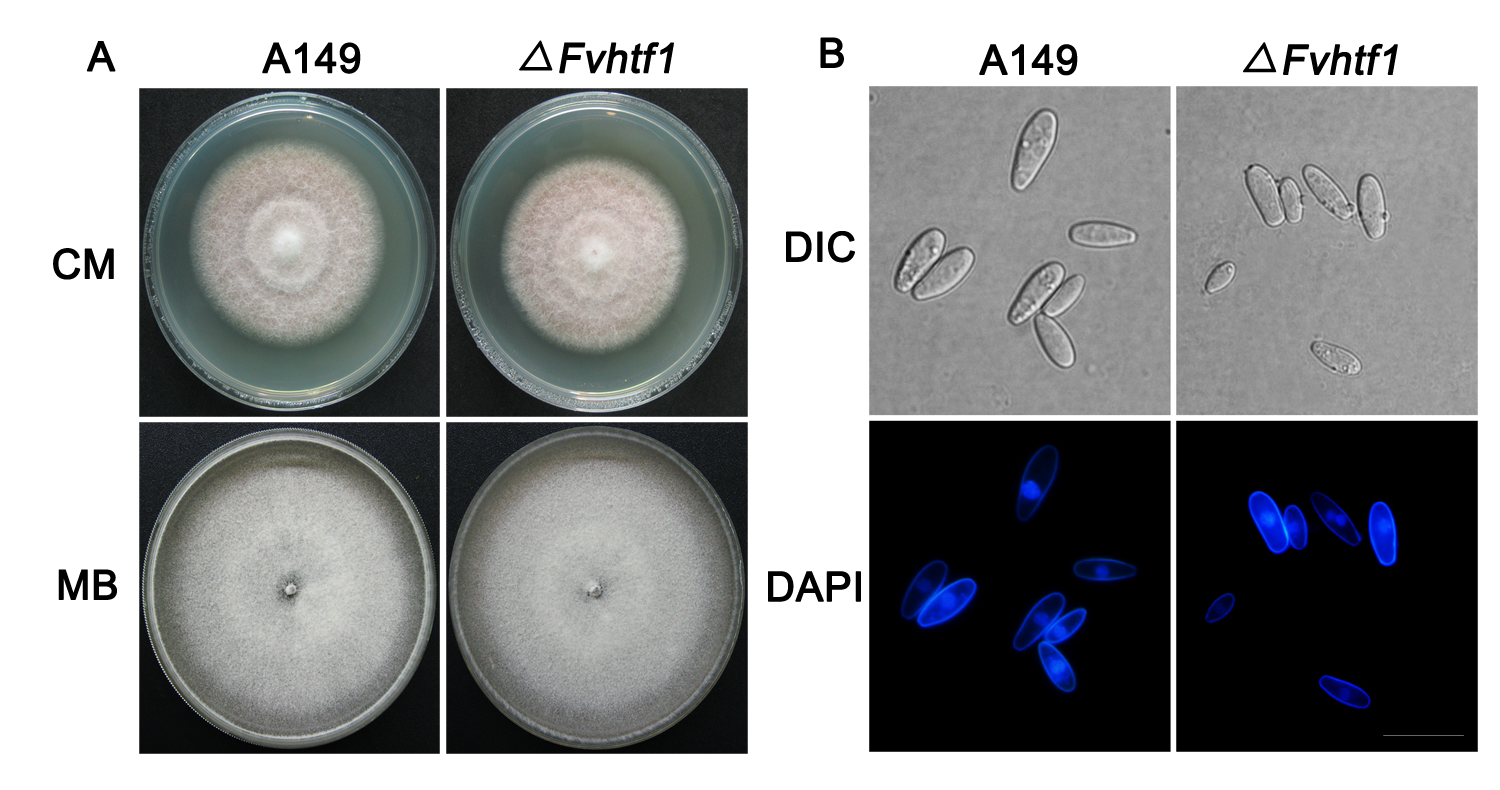

Supplement: Figure S5 — Colony and microconidia morphology of F. verticillioides wild type (A149) and ΔFvhtf1 mutant. (A) Colony morphology of A149 and ΔFvhtf1 mutant grown on CM and MB agar for 6 and 7 days, respectively. (B) Microconidia stained with 4′6-diamidino-2-phenylindole (DAPI) observed under a fluorescence microscope. Bar = 10 µm. (TIFF) [file pone.0045432.s007.tiff]

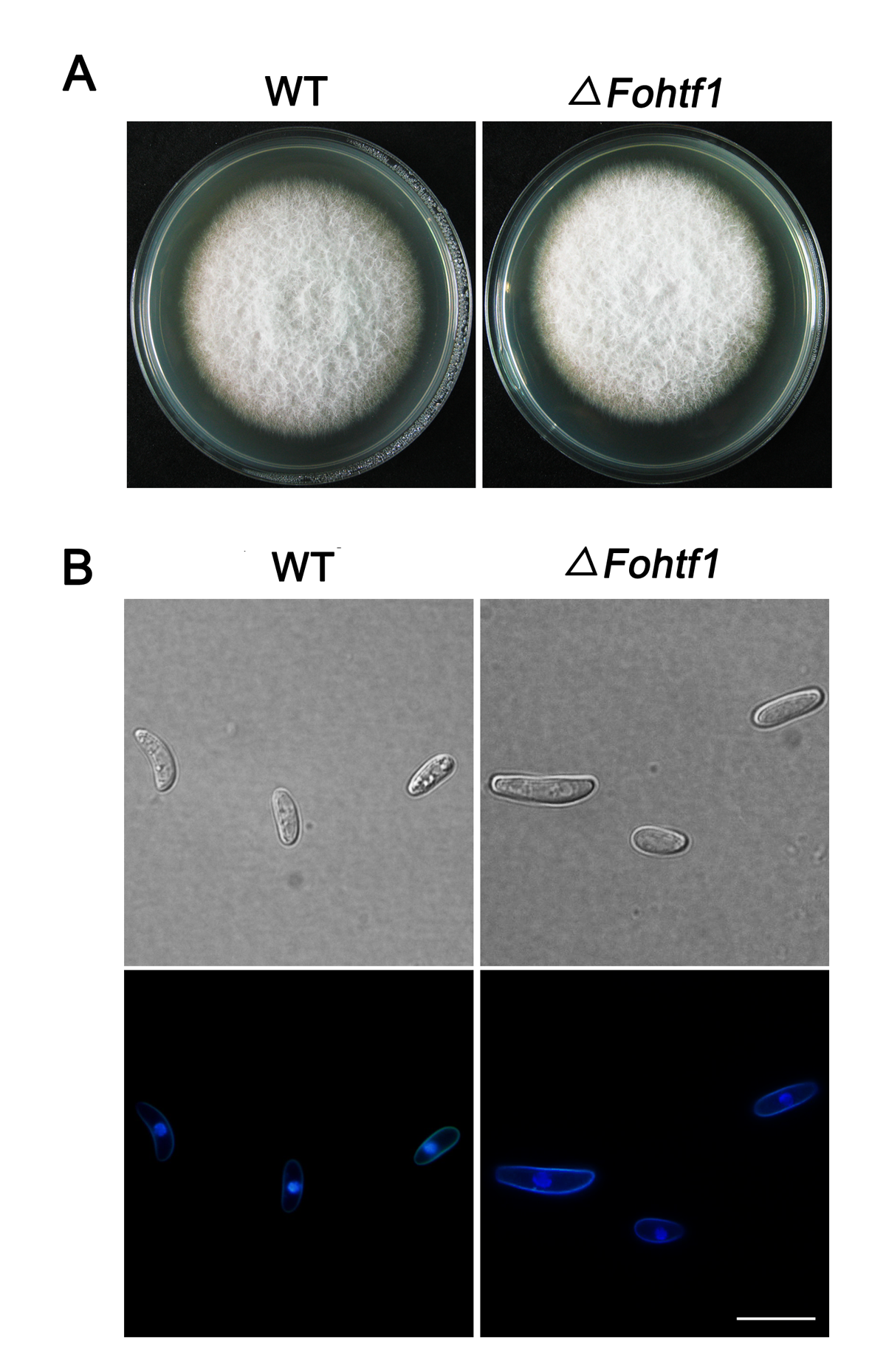

Supplement: Figure S6 — F. oxysporum FoHTF1 gene-replacement strategy and confirmation. (A) Schematic diagram of the genomic region of the FoHTF1 and hph genes. Primers F1 (FO01706AF), R1 (FO01706AR), F2 (FO01706BF) and R2 (FO01706BR) were used to generate FoHTF1 gene replacement constructs. Probe was used to screen and verify gene replacement mutants. N, NcoI. (B) DNA gel blots of NcoI-digested genomic DNA were hybridized with probe. WT, wild-type F. oxysporum. ΔFohtf1-3, ΔFohtf1-6, ΔFohtf1-9 and ΔFohtf1-11, null mutants. ΔFohtf1-Ect, ectopic strain. (TIFF) [file pone.0045432.s008.tiff]

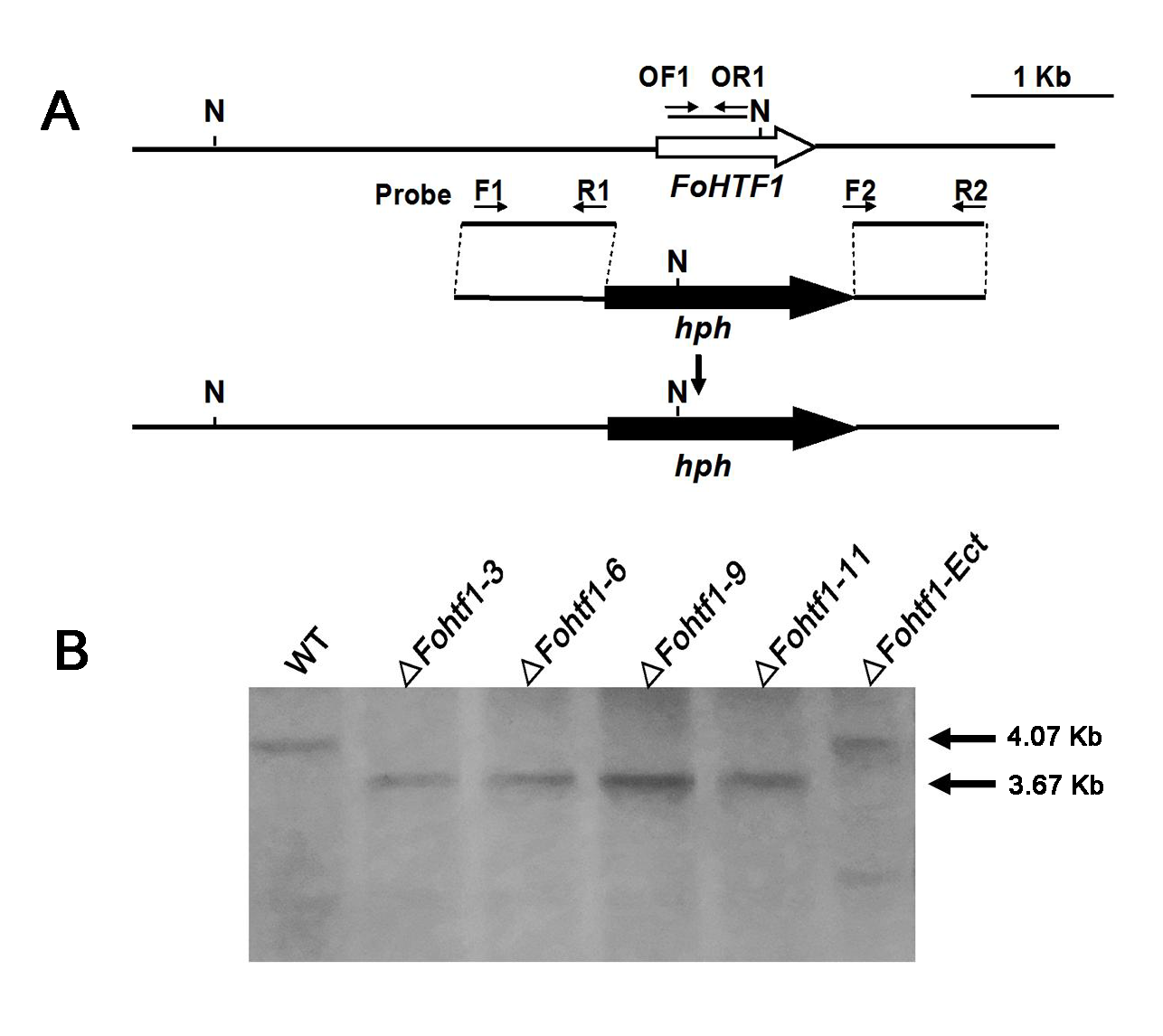

Supplement: Figure S7 — Colony and microconidia morphology of F. oxysporum wild type (WT) and ΔFohtf1 mutant. (A) Colony morphology of WT and ΔFohtf1 mutant grown on CM agar for 6 days. (B) Microconidia stained with 4′6-diamidino-2-phenylindole (DAPI) observed under a fluorescence microscope. Bar = 10 µm. (TIFF) [file pone.0045432.s009.tiff]
